# Computational insights for the hydride transfer and distinctive roles of key residues in cholesterol oxidase

**DOI:** 10.1038/s41598-017-17503-x

**Published:** 2017-12-08

**Authors:** Li-Juan Yu, Emily Golden, Nanhao Chen, Yuan Zhao, Alice Vrielink, Amir Karton

**Affiliations:** 1 School of Molecular Sciences, The University of Western Australia, Perth, WA 6009 Australia; 2 The Key Laboratory of Natural Medicine and Immuno-Engineering, Henan University, Kaifeng, 475004 China; 3 School of Pharmaceutical Sciences, Sun Yat-sen University, Guangzhou, 510006 China; 4 Department of Chemistry, University of California, Davis, California, 95616 United States

## Abstract

Cholesterol oxidase (ChOx), a member of the glucose-methanol-choline (GMC) family, catalyzes the oxidation of the substrate *via* a hydride transfer mechanism and concomitant reduction of the FAD cofactor. Unlike other GMC enzymes, the conserved His447 is not the catalytic base that deprotonates the substrate in ChOx. Our QM/MM MD simulations indicate that the Glu361 residue acts as a catalytic base facilitating the hydride transfer from the substrate to the cofactor. We find that two rationally chosen point mutations (His447Gln and His447Asn) cause notable decreases in the catalytic activity. The binding free energy calculations show that the Glu361 and His447 residues are important in substrate binding. We also performed high-level double-hybrid density functional theory simulations using small model systems, which support the QM/MM MD results. Our work provides a basis for unraveling the substrate oxidation mechanism in GMC enzymes in which the conserved histidine does not act as a base.

## Introduction

Cholesterol oxidase (ChOx) is a flavoenzyme that catalyzes the oxidation of cholesterol to 5-cholesten-3-one and the subsequent isomerization to form the final 4-cholesten-3-one product. The enzyme from *Streptomyces* is a member of the glucose-methanol-choline (GMC) family and contains a single flavin adenine dinucleotide (FAD) cofactor that is non-covalently bound to the protein. Apart from its physiological functions in bacterial metabolism, pathogenesis, and macrolide biosynthesis^1^, ChOx has also been employed as a useful biotechnological tool, for example, for the determination of serum cholesterol levels^2,3^.

**Figure 1:**
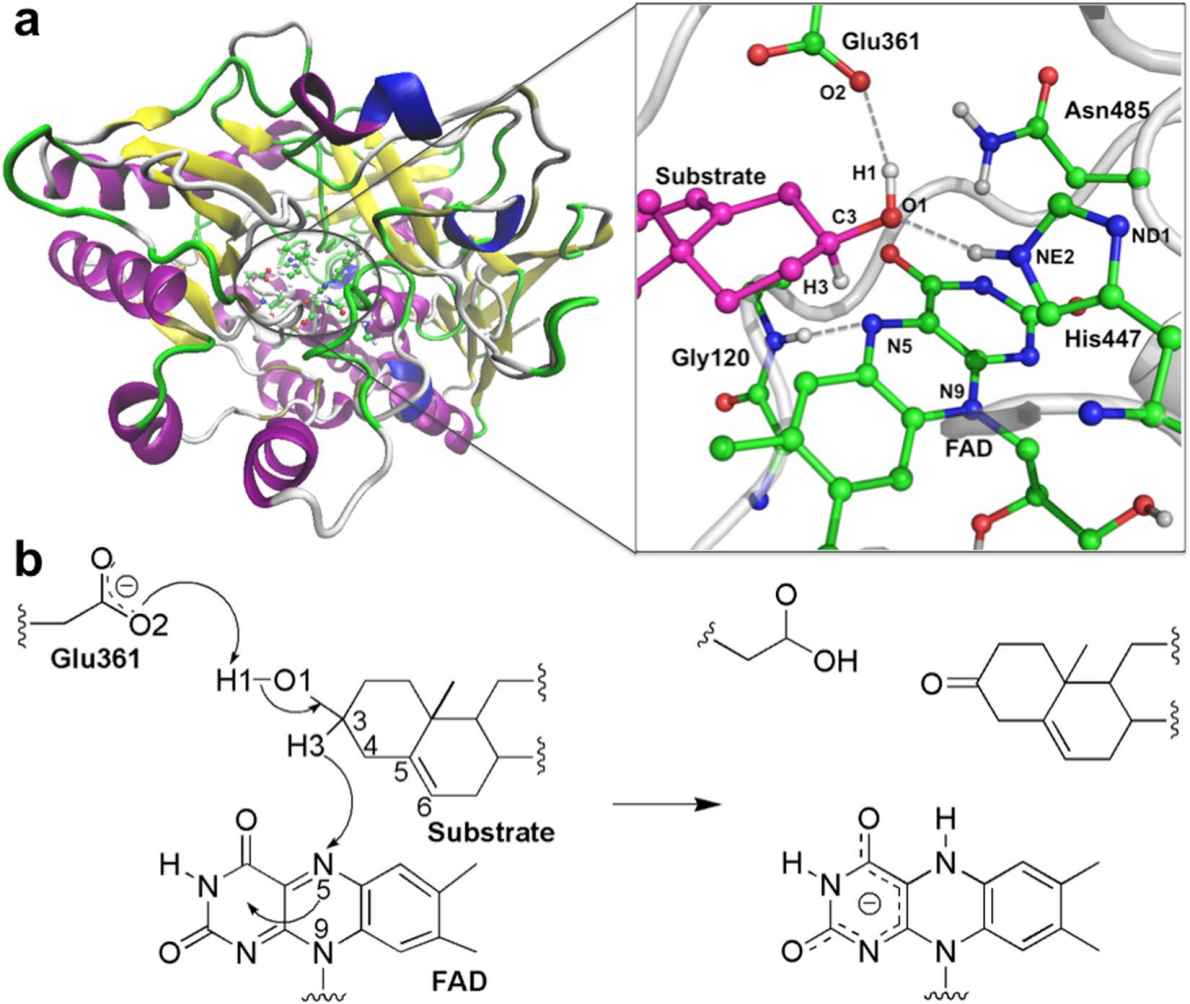
Key residues in the active site of ChOx and proposed reaction mechanism. (**a**) The substrate is colored in magenta. The grey dashed lines represent H-bond interactions. Atomic color scheme: H, white; C, green; O, red; N, blue. (**b**) Proposed mechanism for the substrate oxidation reaction catalyzed by ChOx.

A number of experimental studies investigated the structure and catalytic mechanism of ChOx over the past two decades^4–11^. The active site of ChOx is shown in Fig. 1a. With regard to the enzymatic mechanism, Sampson *et al*.^7–11^ identified three residues (Glu361, His447, and Asn485) that are significant for the enzymatic activity and Vrielink *et al*.^4,5^ revealed that Gly120 also plays an important role in the catalytic mechanism.

Mutations of the Glu361 residue in the active site to glutamine and aspartate result in 31-fold^9^ and 14-fold^8^ reductions, respectively, in the *k*_cat_ value for the oxidation of the substrate compared to the wild-type (WT) enzyme. This suggests that the carboxylic acid residue at this position may be important for the oxidative activity^6^. The His447 residue is located in the active site and the NE2 hydrogen forms a hydrogen bond with the hydroxyl group of the substrate. Mutations of His447 to glutamine and asparagine result in 140-fold and 4400-fold reductions in the *k*_cat_ value respectively^9^, relative to the WT enzyme, suggesting that His447 plays a significant role in the catalytic activity. However, the double mutant, His447Gln/Glu361Gln, exhibits a 500-fold decrease in the *k*_cat_ value relative to the WT enzyme^10^, which was 3-fold slower than that for the His447Gln single-mutant. These mutagenesis results indicate that factors affecting the oxidation rate of the two separate mutants, His447Gln and Glu361Gln, are not entirely additive and may be the result of structural perturbations rather than the absence of an active site base. We note that the double mutation does not completely prevent the hydride transfer implying that possibly the FAD is sufficiently electrophilic to oxidize the substrate without complete proton abstraction^6^. In addition, other structural studies have suggested that Asn485 forms an N–H•••π electrostatic interaction with the flavin π-system to facilitate reduction of the FAD^11^. Mutation of Asn485 to leucine decreases the *k*_cat_ value as well. It has been proposed that this residue modulates the electrostatic potential of the flavin to enhance substrate oxidation^11^. The Gly120 residue, which is positioned below the isoalloxazine ring system and functions as a hydrogen bond donor to the N5 atom of FAD, was also proposed to be important in orienting the orbitals of the N5 atom of the FAD cofactor, thus priming it for the hydride transfer chemistry^4,5^.

The recent high resolution X-ray structure of the oxidized ChOx in the presence of an isopropanol substrate (PDB ID: 4U2T)^4^ enables us to build a model of the ChOx active site composed of Glu361, the FAD cofactor, and the steroid substrate (see Fig. 1b). It was proposed that ChOx catalyzes the oxidation of the substrate *via* a hydride transfer from the steroid substrate C3 atom to the N5 atom of FAD and concomitant reduction of the FAD cofactor^5,6^. Despite extensive experimental work on the structure of ChOx, a detailed theoretical simulation of the catalytic mechanism and roles of the key residues in the active site was not carried out. In this work, we performed extensive combined quantum mechanics and molecular mechanics molecular dynamics (QM/MM MD) simulations including the catalytic mechanism and substrate binding, and high-level double-hybrid density functional theory (DHDFT) calculaions to explore the enzymatic catalysis. The results from the DHDFT calculations using small model systems support the QM/MM MD results. The combination of these two approaches gives an in-depth understanding of flavoenzyme catalysis by ChOx.

## Results

### QM/MM MD simulations on enzymatic mechanism

#### Catalytic mechanism of ChOx

**Figure 2:**
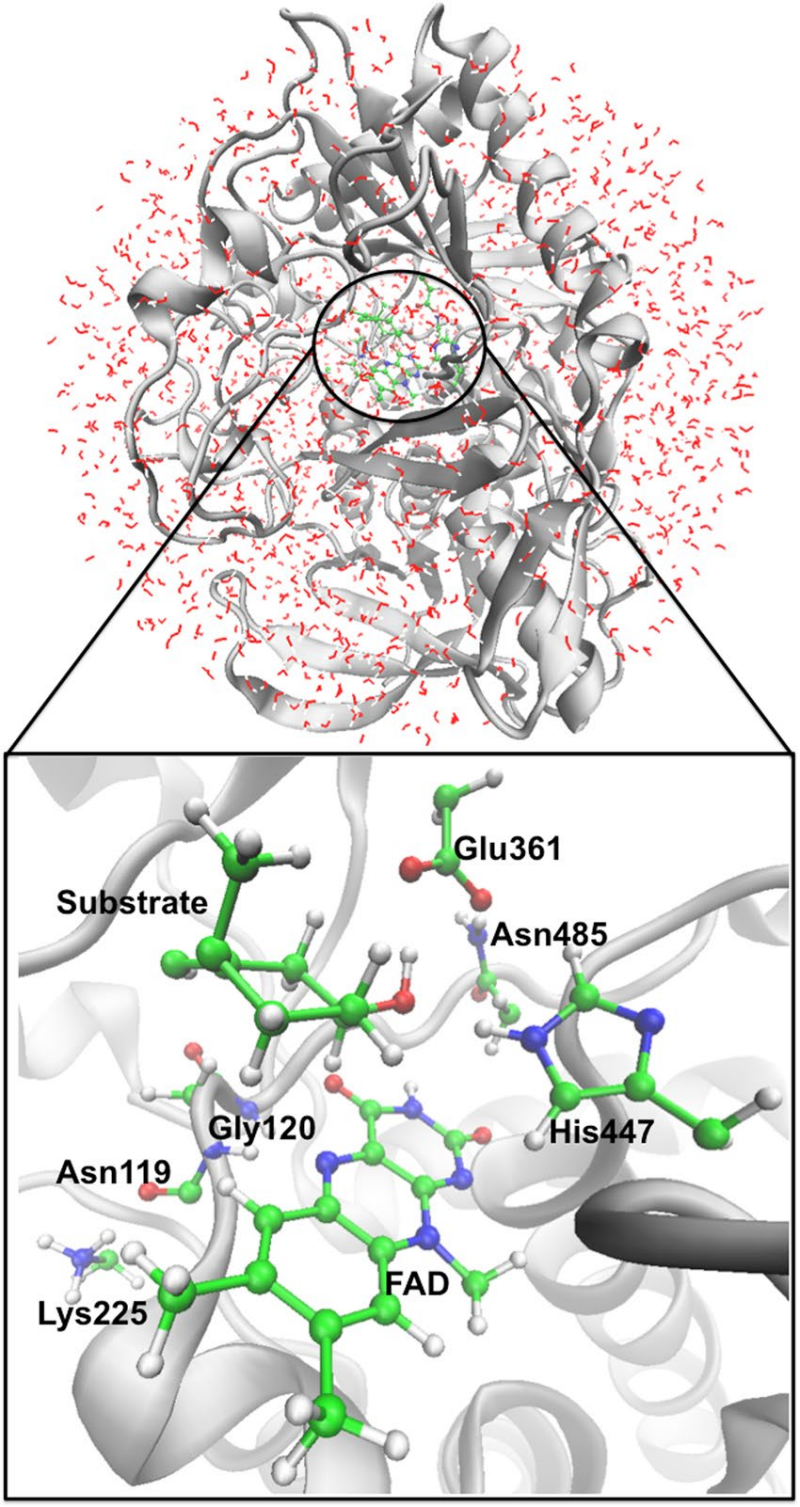
Overall view of the QM/MM model for ChOx and the active site. The active site includes the Lys225, Asn119, Gly120, Glu361, Asn485, and His447 residues as well as FAD and the substrate. The residues in the QM region are shown as ball and stick, the protein as ribbons, and the solvent as wireframes. The QM/MM boundary settings are specified in the subsection of “QM/MM MD simulation”.

10 ns molecular dynamics (MD) simulations for the enzyme-substrate complex model were performed. The root-mean-square deviation (RMSD) of the protein backbone is indicated in Supplementary Fig. S1. The last snapshot of MD simulations was chosen as the initial QM/MM model by removing the water molecules beyond 30 Å from the N9 atom of the FAD cofactor (see Fig. 2). We considered two different reaction coordinates (RC1 and RC2, see Supplementary Fig. S2). The reaction coordinate RC1 is defined as the difference between the C3–H3 and N5–H3 bond distances that are involved in the hydride transfer: RC1 = d_C3–H3_ – d_N5–H3_. The reaction coordinate RC2 is defined as RC2 = d_C3–H3_ – d_N5–H3_ – d_O2–H1_, that is it describes the movement of both the hydride and the proton. The relative energy barrier heights for RC1 and RC2 are 130.6 and 125.4 kJ mol^−1^, respectively. Another way of describing the combined movement of both the hydride and the proton is to fix the length of the O1–H1 distance at certain values and to scan along the reaction coordinate RC1. In particular, the O1–H1 distance was fixed at 1.01, 1.06, 1.11, 1.16, 1.21, 1.26, 1.31, 1.36, 1.41, 1.46, 1.51, 1.56, 1.61, 1.66, 1.71, and 1.76 Å. This is a cost-effective way to approximate a 2D scan of both the hydride and proton transfers. This approximated 2D scan is shown in the Supplementary Fig. S3a. It clearly shows that a first-order saddle point is obtained for the hydride transfer for each of the fixed O1–H1 bond distances. We find that the lowest reaction barrier height of 128.4 kJ mol^−1^ is obtained at r(O1–H1) ≈ 1.56 Å (Supplementary Fig. S3b). This result is consistent with our results for RC1 and RC2 above. Considering the computational cost of the potential of mean force (PMF) calculations, we chose RC1 as our final reaction coordinate since the reaction barrier height obtained along this coordinate is in good agreement with our best reaction barrier height obtained from the approximate 2D scan. By using QM/MM MD simulations and umbrella sampling, the free energy profile and corresponding configurations of reactant, transition state, and product involved in the enzymatic catalysis have been determined. In order to verify the efficiency of our sampling, the configurational overlap in the range of 5 ps to 20 ps is shown in Supplementary Fig. S4. This figure shows that the sampling covers the whole reaction coordinate with the current step size and biasing harmonic potential.

**Figure 3:**
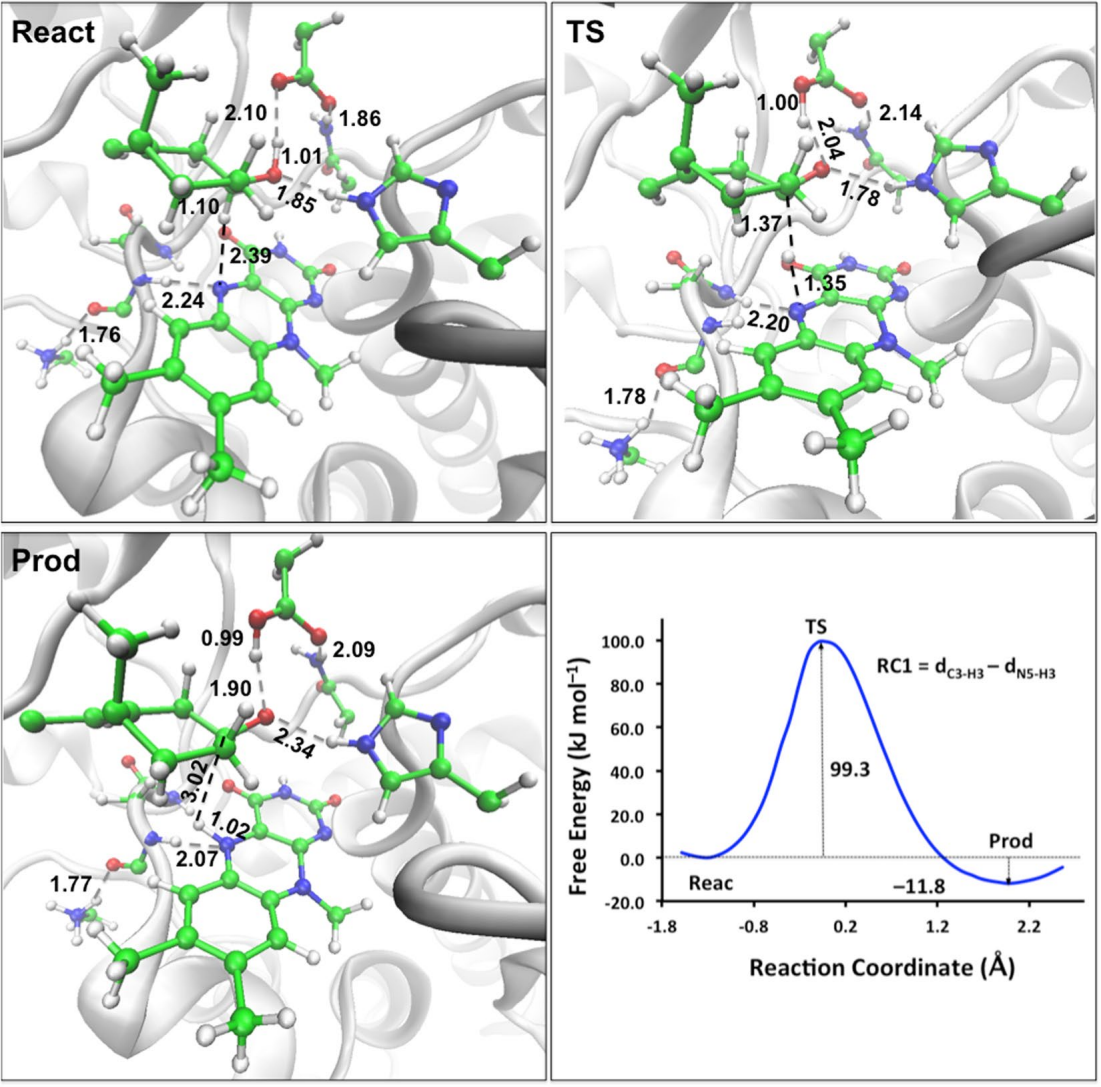
Structures of reactant (**React**), transition state (**TS**), product (**Prod**), and free energy profile of the hydride transfer in ChOx. Hydrogen bonds are shown as grey dashed lines. The bonds being broken and formed are shown as black dashed lines. The average distances (in Å) for selected bonds are taken from umbrella sampling.

Figure 3 shows the free energy profile and the structures of the reactant (**React**), transition structure (**TS**), and product (**Prod**) obtained from the umbrella sampling technique. It is noteworthy that the deprotonation of the substrate by Glu361 occurs synchronously with the hydride transfer (see **TS** in Fig. 3). The O1–H1 bond distance increases from 1.01 ± 0.03 (**React**) to 1.90 ± 0.17 Å (**Prod**), while the O2–H1 bond distance decreases from 2.10 ± 0.45 (**React**) to 0.99 ± 0.01 Å (**Prod**). The free energy barrier of the hydride transfer is 99.3 kJ mol^−1^, and the reaction energy is exergonic by 11.8 kJ mol^−1^. In the QM/MM transition structure, the length of the C3–H3 bond that is being broken is 1.37 ± 0.07 Å and the length of the N5–H3 bond that is being formed is 1.35 ± 0.09 Å. In this transition structure the O1–H1 and O2–H1 bond distances are 2.04 ± 0.30 and 1.00 ± 0.03 Å, respectively. This indicates that the proton has transferred before the hydride transfer has been completed. We note that we cannot directly compare the barrier obtained from the QM/MM MD simulations with previous experimental kinetic data^9^ since the kinetic measurements do not reflect the rate of the hydride transfer alone. However, the experimental *k*_cat_ value obtained for the wild type (44 ± 2 s^−1^) indicates that our QM/MM MD free energy barrier for the hydride transfer likely represents an overestimation. Nevertheless, it is important to stress that the present work is concerned largely with relative reaction barrier heights (that will benefit from some cancellation of errors) and are likely to be more accurate than the absolute reaction barrier heights.

**Figure 4:**
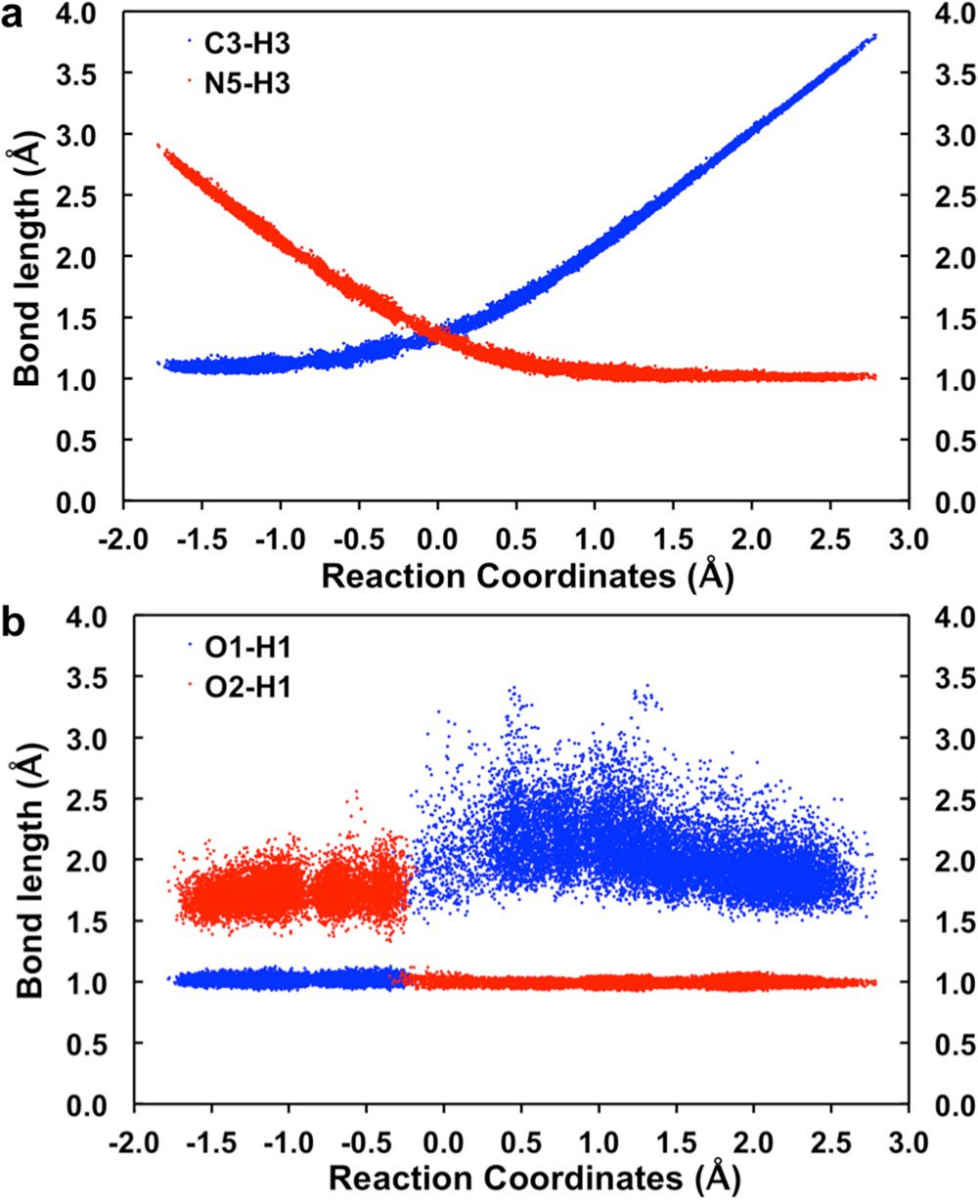
Statistics for bond lengths from the snapshots along RC1. (**a**) the C3–H3 and N5–H3 (**b**) the O1–H1 and O2–H1 bond lengths.

In order to clarify the order of the proton and hydride transfers in the catalytic process, the entire snapshots along RC1 have been collected. Figure 4 plots the snapshots for the C3–H3, N5–H3, O1–H1, and O2–H1 distances along the reaction coordinate RC1 (between the interval RC1 = −1.59 to 2.61 Å). As shown in Fig. 4a, the length of the C3–H3 distance increases smoothly from ~1.1 to 3.8 Å, while the length of the N5–H3 decreases from ~2.9 to 1.0 Å along RC1. The cross-point of these two lines corresponds to the **TS** shown in Fig. 3. At this point, the C3–H3 and N5–H3 distances are equal to one another (i.e., RC1 = 0.0 Å). Figure 4b shows that the proton transfer from the hydroxyl group of the substrate to the oxygen (O2) of Glu361 occurs at RC1 ≈ −0.2 Å. More specifically, the O1–H1 bond length remains at ~1.0 Å and the O2•••H1 distance fluctuates at ~1.8 Å along RC1 from around RC1 = −1.7 to −0.2 Å. The atom H1 jumps to O2 at RC1 ≈ −0.2 Å, after which the O2–H1 bond length stays at ~1.0 Å and the O1•••H1 length fluctuates at ~ 2.3 Å. These results indicate that the proton transfer occurs spontaneously with the hydride transfer.

The bond lengths of His447NE2H–O1 and LysNH_3_–O were also monitored during the hydride transfer (see Supplementary Fig. S5). The hydrogen bond distance between the NH_3_ group of Lys225 and the O=C group of Asn119 (labeled as LysNH_3_–O in Supplementary Fig. S5) fluctuates at ~1.8 Å during the hydride transfer, which implies that this hydrogen bond has a negligible effect on the barrier height for the hydride transfer. As for the hydrogen bond distance between His447 and the hydroxyl group of the substrate, labeled as NE2H•••O1, it is ~1.8 Å before the proton transfer (RC1 = –0.2 Å) and increases to ~2.6 Å after the proton transfer. This indicates that the His447 plays a significant role during the oxidation reaction. The hydrogen bond formed between His447 and the hydroxyl group of the substrate orients the hydroxyl H atom towards the Glu361 base, which facilitates the proton transfer. This also results in an energetically favorable *trans* arrangement between the substrate hydroxyl H atom and the hydride.

#### The effects of mutating His447 on the active site environment

**Figure 5:**
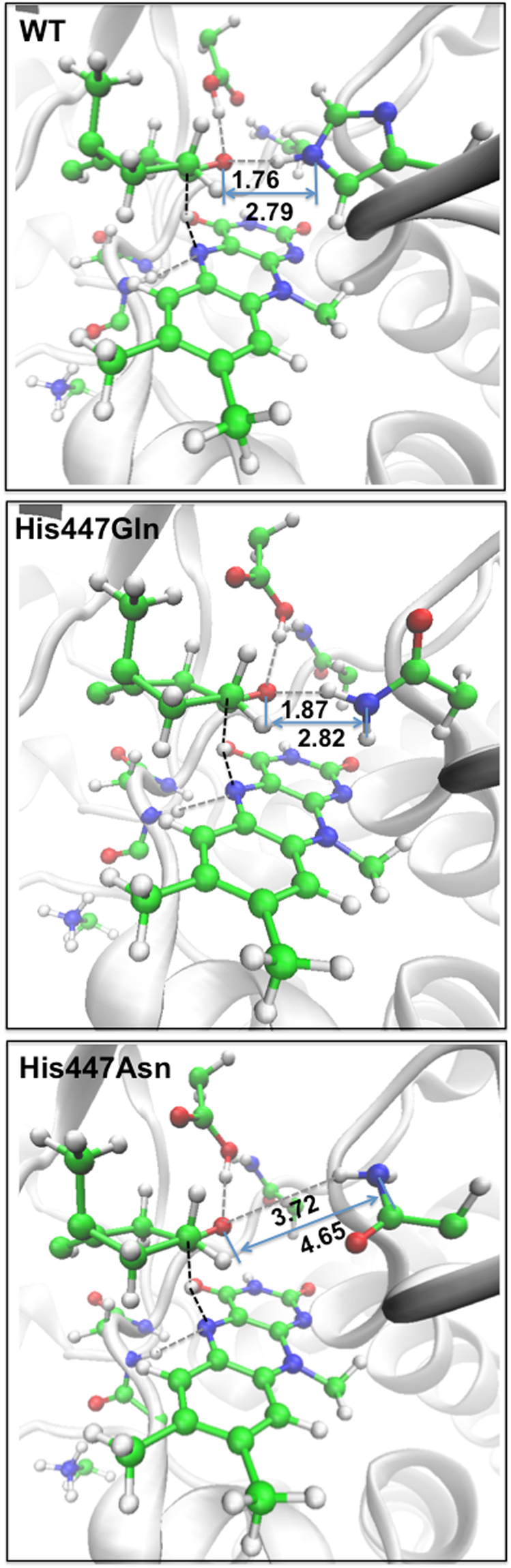
Transition structures for the WT, His447Gln, and His447Asn systems obtained by means of QM/MM scans. Hydrogen bonds are shown as grey dashed lines. The bonds being broken and formed are shown as black dashed lines. The distances for selected bonds are given in Å.

Previous experimental point mutations revealed that the His447 residue plays an important role in the oxidation reaction of the substrate in ChOx^9^. For example, mutations of the His447 residue to glutamine and asparagine result in 140-fold and 4400-fold reductions, respectively, in the *k*_cat_ value for the oxidation reaction^9^. However, the atomic resolution crystal structure showed that the NE2 atom of His447 was protonated^6^. This led to the suggestion that His447 is not a catalytic base. To further elucidate the role of His447, QM/MM calculations have been performed on systems in which the His447 residue has been mutated to glutamine and asparagine (denoted by His447Gln and His447Asn, respectively). In these computational experiments, we carried out QM/MM energy scans along the reaction coordinate of RC1 using the QM/MM optimized structures to obtain the minimum energy reaction path (see Supplementary Fig. S6). However for reasons of computational cost, the subsequent QM/MM free energy perturbation and 20 ps QM/MM MD simulations with umbrella sampling were not performed in this section. Figure 5 gives the **TS**s of the WT, His447Gln, and His447Asn systems. The energy barrier height for the hydride transfer in the mutated systems is higher by 32.8 (His447Gln) and 66.3 kJ mol^−1^ (His447Asn) relative to that in the WT system (as shown in Supplementary Fig. S6). These QM/MM scan results are consistent with the mutagenesis experiments, where mutation of His447 to asparagine results in a larger reduction in the *k*_cat_ value compared to the glutamine mutant.

The selected bond distances for the **TS**s of the WT, His447Gln, and His447Asn systems are also labeled in Fig. 5. In the QM/MM-optimized structures the hydrogen bond distances between O1 of the substrate and NE2H of His447, His447Gln, and His447Asn are 1.76 (WT), 1.87 (His447Gln), and 3.72 Å (His447Asn). As we discussed earlier, the hydrogen bond formed between His447 and the hydroxyl group of the substrate facilitates the proton transfer. When the His447 residue is mutated to asparagine, due to the shorter side chain in asparagine relative to histidine, a hydrogen bond to the substrate is not formed (i.e., the distance between O1 and NE2H is 3.72 Å). These results indicate that the weak hydrogen bond or lack of hydrogen bond interactions between the substrate and the key residue (i.e. His447) may impede the proton transfer that facilitates the hydride transfer in the WT enzyme, and further increases the barrier height for the hydride transfer. This could be due to the fact that the OH group of the substrate is not as well fixed in the active site and the catalytic environment has changed in the mutagenesis systems (i.e. His447Gln and His447Asn).

In order to consider the protein/water environment for the His447Gln mutant system, we carried out 20 ns MD and QM/MM MD simulations followed by a potential energy scan. Firstly, 20 ns MD simulations were performed for the His447Gln mutant and a stable mutant was obtained. The RMSD of the protein backbone is shown in Supplementary Fig. S8a. Then the QM region was chosen similarly to that of the wild-type and optimized at B3LYP/6–31 G(d) level of theory. In order to obtain a stable protein/water system, before running the potential energy surface scan, we ran 5 ps QM/MM MD for the His447Gln mutant system. Then we carried out the relative energy scans using the different reaction coordinates (RC1 and RC2, *vide supra*). The scan plots are displayed in Supplementary Fig. S9. The relative energy barrier heights obtained from RC1 and RC2 are 158.7 and 162.8 kJ mol^−1^ respectively, which are in good agreement with the one shown in Supplementary Fig. S6. We believe that these relative energy barrier heights demonstrate the important role that His447 plays in the enzyme. We note that the main purpose of this work is to show that the predicted theoretical trend of the barrier height when mutating His447 to Gln is consistent with the experimental reduction of the *k*_cat_ value, rather than to reproduce the experimental *k*_cat_ value, which would require extensive additional PMF calculations.

Similar to our approach with the wild-type, we confirm that the chosen reaction coordinates (RC1 and RC2) give results that are consistent with the results of the more rigorous approximated 2D scan for the His447Gln system. In particular, the O1–H1 distance was fixed at 0.98, 1.03, 1.08, 1.13, 1.18, 1.23, 1.28, 1.33, 1.38, 1.43, 1.48, 1.53, 1.58, 1.63, 1.68, 1.73, 1.78, 1.83, 1.88, 1.93, 1.98, 2.03, 2.08, 2.13, and 2.18 Å for each scan of the hydride transfer reaction coordinate. The 2D scan is given as Supplementary Information (Supplementary Fig. S11a). This figure shows that a first-order saddle point is obtained for the hydride transfer for each of the fixed O1–H1 bond distances. We find that the lowest reaction barrier height of 156.8 kJ mol^−1^ is obtained at r(O1–H1) ≈ 1.63 Å (Supplementary Fig. S11b). This result is consistent with our results for RC1 and RC2 above. Thus, we conclude that both the RC1 and RC2 reaction coordinates can efficiently describe the hydride transfer as well as the proton transfer. Considering the computational cost of the PMF calculations and the main purpose of this work, we chose RC1 as our final reaction coordinate for the potential energy scan.

#### The role of key residues in substrate binding

It has been proposed that the hydrogen-bonding networks involving Gly120, Glu361, His447, Asn485, and the substrate play important roles in substrate binding^4,8,9,11,12^. It is interesting to evaluate the binding free energy and the interaction between the substrate and the key residues. The ligand-residue interaction for residues from 101 to 499 in ChOx and binding free energy decomposition in the active site are displayed in Supplementary Fig. S7. We note that Gly120, Glu361, His447, and Asn485 play important roles in stabilizing the substrate in the active site (see Supplementary Fig. S7a). It is noteworthy that other residues also play significant roles in the binding free energy, e.g., Met114, Met122, Val250, Pro364, Tyr446, Pro448, and Phe487. For example, they are associated with binding free energies ranging from −5.2 (Pro364) to −19.0 kJ mol^−1^ (Pro448). These residues are involved in the surrounding hydrogen bond network of the FAD cofactor and stabilize FAD in the active site. However, they are not directly associated with stabilizing the substrate in the active site.

In order to get additional insights into individual contributions from the Glu361, His447, and Asn485 residues to the substrate binding, the binding free energies were calculated for the WT and the Glu361Ala, His447Ala, and Asn485Ala mutant systems using the MM-GBSA method^13,14^. The Gibbs free binding energies for the WT and mutant systems as well as the differences between them are listed in Table 1. The Gibbs free binding energies for the mutants are lower than that for the WT by: 35.8 (Glu361Ala), 41.3 (His447Ala), and 4.8 kJ mol^−1^ (Asn485Ala). Mutations of these key residues result in destabilization of the substrate in the reaction center. Thus, the catalytic process becomes less efficient. This is another indication of the significant roles of these key residues in stabilizing the substrate in the active site. In order to obtain further insights into the roles of key residues in the enzymatic catalysis we also carried out double-hybrid density functional theory (DFT) calculations, which will be discussed in the following section.

**Table 1:** Gibbs binding free energies (∆*G*_bind_, in kJ mol^−1^) for the WT, Glu361Ala, His447Ala, and Asn485Ala systems as well as the differences between the WT and mutant systems (∆∆*G*_bind_) calculated with the MM-GBSA method.

System	∆*G*_bind_^*a*^	∆∆*G*_bind_
WT	−62.5	0.0
Glu361Ala	−27.0	−35.8 ± 6.3
His447Ala	−21.3	−41.3 ± 6.4
Asn485Ala	−57.7	−4.8 ± 1.5

^a^The Gibbs binding free energy (∆*G*_bind_) contains electrostatic, van der Waals, polar, non-polar, and entropy contributions.

### Double-hybrid DFT calculations for probing the effects of hydrogen-bonding interactions on the barrier height of the hydride transfer

Double-hybrid density functional theory (DHDFT) calculations were performed in order to obtain insights into the roles of the residues in the catalytic mechanism of the hydride transfer from the C3 atom of the substrate to the N5 atom of the FAD cofactor. These computational experiments were carried out on a number of model systems with increasing size in order to elucidate the catalytic roles of the Glu361 and Gly120 residues on the barrier height for the hydride transfer. In particular, we consider the following models. Model **A** consists of the FAD cofactor (modeled by an isoalloxazine ring system) and the steroid substrate (modeled by an HOCHMe_2_ moiety). Model **B** consists of an additional Glu361, which forms a strong hydrogen bond with the hydroxyl group of the steroid substrate. Model **C** includes an additional Gly120 (modeled by a dimethylamine molecule), which forms a hydrogen bond to the N5 atom of FAD. The atoms involved in these three models are displayed in Supplementary Fig. S12.

**Figure 6:**
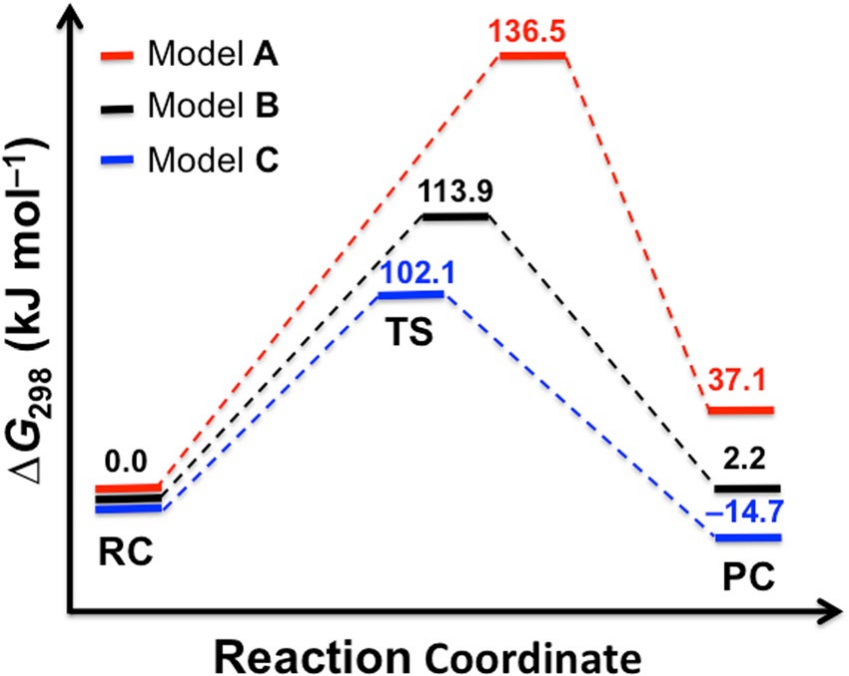
Gibbs free energy profiles for the hydride transfer in models A–C calculated at the RI-B2GP-PLYP-D3/Def2-QZVPP level of theory. Model A includes FAD and the substrate (red line); model B includes FAD, the substrate, and Glu361 (black line); and model C includes FAD, the substrate, Glu361, and Gly120 (blue line). The transition structures (TSs) involved in these reactions are shown in Fig. 7. The corresponding reactant complexes (RCs) and product complexes (PCs) are shown in Supplementary Fig. S13.

**Figure 7:**
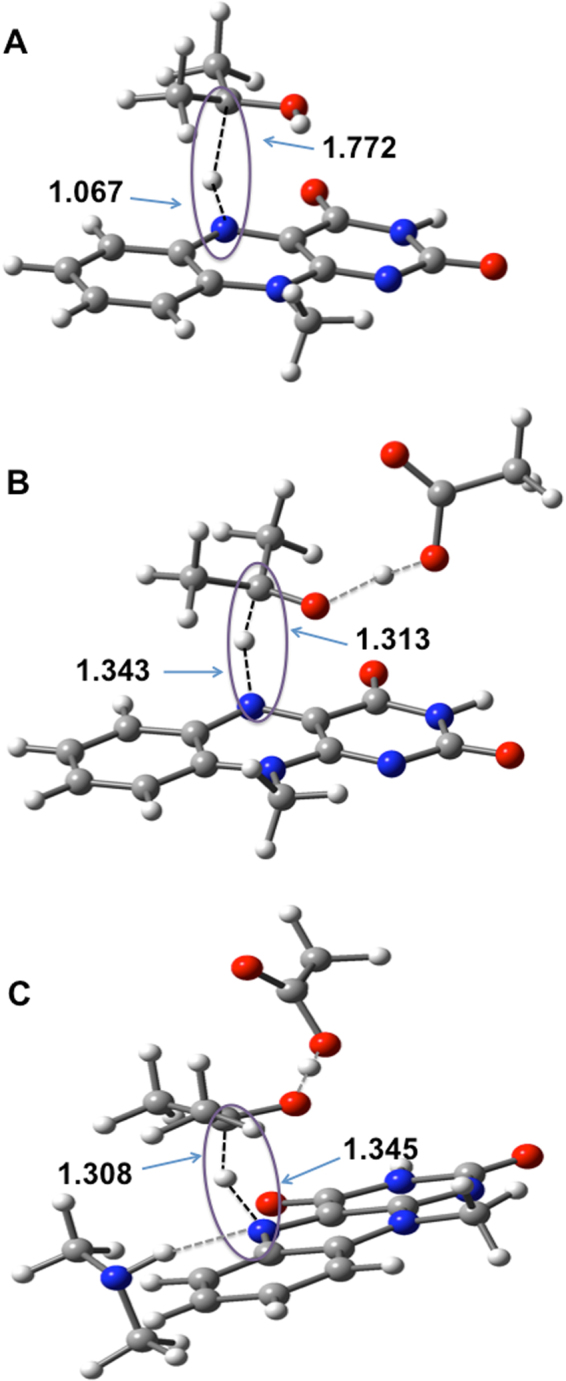
B3LYP-D3/6–31+G(2df,p) optimized TSs located on the potential energy profiles in models **A**,** B**, and **C**. Hydrogen bonds are shown as grey dashed lines. The bonds that are broken and formed in the TSs are shown in black dashed lines with purple circles. The C3–H3 and H3–N5 bond distances are given in Å. Atomic color scheme: H, white; C, grey; N, blue; O, red.

Figure 6 depicts the Gibbs free energy profile (∆*G*_298_) for the hydride transfer in models **A** (red), **B** (black), and **C** (blue). The calculated reaction barriers (∆*H*_298_^‡^) and enthalpies (∆*H*_298_) are listed in Supplementary Table S1. Figure 7 shows the optimized transition structures (TSs) involved in models **A**–**C**. The corresponding reactant complexes (RCs) and product complexes (PCs) are shown in Supplementary Fig. S13.

Model **A**, which consists of the FAD cofactor and the substrate, is the simplest binary system for modeling the hydride transfer. The hydride transfer in this model is expected to be both kinetically and thermodynamically unfavorable, however we start with this simple picture since it provides a reference point for the effects from the additional residues in models **B** and **C**. For the hydride transfer in model **A**, we obtain an activation Gibbs free energy of ∆*G*_298_^‡^ = 136.5 kJ mol^−1^ and the reaction is endergonic by 37.1 kJ mol^−1^. According to the Hammond–Leffler postulate the high reaction barrier and energy suggest a late TS^15^. Indeed, in the TS, the hydride transfer between the C3 and N5 atoms is almost complete, the C3•••H^−^ and ^−^H•••N5 distances being 1.772 and 1.067 Å, respectively. For comparison, the ^−^H•••N5 bond length in the product complex is 1.035 Å (see Supplementary Fig. S13).

The enzymatic role of Glu361 is to act as a base to abstract the proton from the hydroxyl group of the substrate. Abstracting the hydroxyl proton has two effects on the reaction profile for the hydride transfer: (i) it activates the hydride transfer by pushing electron density to the C3–H3 bond and (ii) it stabilizes the product by converting the isopropanol carbocation to a neutral acetone. Model **B** includes the Glu361 residue. Indeed, the activation free energy in this model is lower by 22.6 kJ mol^−1^ relative to that in model **A**. In the TS of model **B**, the hydride transfer from C3 to N5 is concomitant with the proton transfer from the substrate to Glu361 (see model **B** in Fig. 7). In this TS, the hydride is located halfway between the C3 and N5 centers, in particular we obtain bond distances of 1.313 (C3•••H^–^) and 1.343 Å (^–^H•••N5). These bond distances indicate that the TS for the hydride transfer in model **B** is earlier than that in model **A**, and therefore the lower barrier is consistent with the Hammond–Leffler postulate^15^. The effect of Glu361 on the barrier height of the hydride transfer is also demonstrated by considering the atomic polar tensor (APT) charges on the hydride and the substrate in the TS^16,17^. In model **A**, the overall charge on the substrate is +0.245 a.u. and the atomic charge on the hydride is −0.178 a.u. However, in model **B**, due to the proton transfer from the substrate to Glu361, the overall charge on the substrate becomes negative (namely it becomes –0.331 a.u.) and the hydride becomes more negatively charged as well with an atomic charge of –0.507 a.u. The partial negative charges on the hydride and the substrate in model **B** should facilitate an easier hydride transfer compared with model **A** where they have opposite charges. Abstracting the hydroxyl hydrogen in model **B** also stabilizes the product by converting the isopropanol carbocation to a neutral acetone. In this situation, the reaction energy is nearly thermo neutral, in particular it is reduced from +37.1 (model **A**) to +2.2 kJ mol^−1^ (model **B**).

It has been previously suggested that the hydrogen bond between the main chain NH of Gly120 and the flavin redox center (N5) may facilitate the catalytic hydride transfer by re-arrangement of the lone-pair electrons on N5^4^. Model **C** includes a dimethylamine (HNMe_2_) molecule that mimics the hydrogen bond between Gly120 and FAD. The optimized TS is shown in Fig. 7 and the optimized O2•••H1 and O1•••H1 distances in the RC, TS, and PC are listed in Supplementary Table S2. The Gibbs free energy barrier height of model **C** is 102.1 kJ mol^−1^, i.e., it is 11.8 kJ mol^−1^ lower than that of model **B** (see Fig. 6), and the reaction energy of this model is exergonic by 14.7 kJ mol^−1^. These values are in excellent agreement with the barrier obtained from the QM/MM MD simulations, namely: ∆*G*_298_^‡^ = 99.3 and ∆*G*_298_ = –11.8 kJ mol^−1^. A natural bond orbital (NBO) analysis^18^ performed at the B3LYP-D3/6–31+G(2df,p) level of theory revealed that lone-pair → σ_N–H_ interactions between the N5 center and N–H bond of Gly120 are associated with a stabilization energy of 20.6 kJ mol^−1^ in the reactant complex and 26.2 kJ mol^−1^ in the transition structure. The greater stabilization energy in the TS relative to the RC may partially account for the catalytic enhancement provided by the Gly120 residue. The above stabilization energies are approximated by the second-order perturbation energy (*E* ^*(2)*^) obtained from the NBO analysis^19^.

In summary, our high-level DHDFT modeling suggests that the Glu361 residue, which abstracts the proton from the hydroxyl group of the substrate, has a significant influence on the Gibbs free energy barrier for the enzymatic hydride transfer. Namely, the Glu361 residue lowers the barrier by 22.6 kJ mol^−1^ (comparison of models **A** and **B**). We also show that the Gly120 residue lowers the barrier further by 11.8 kJ mol^−1^ (comparison of models **B** and **C**).

## Discussion

Extensive QM/MM MD simulations and DHDFT calculations have been carried out in order to explore the mechanism of the oxidation reaction catalyzed by ChOx as well as the roles that the surrounding residues play for the hydride transfer from the substrate to the co-factor.

Using QM/MM MD simulations and umbrella sampling we explored the reaction mechanism for the hydride transfer. In addition, the roles of the key residues in the substrate binding are examined. Point mutations of His447 to glutamine and asparagine increase the relative energy barrier height of the hydride transfer by 32.8 and 66.3 kJ mol^−1^, respectively. These results are in good quantitative agreement with the reduction in the experimental *k*_cat_ values upon mutation. Our simulations suggest that mutation of His447 disrupts the hydrogen-bonding network around the substrate, which in turn results in the substrate being in a less favorable position for the proton transfer from the substrate to the base Glu361. In addition, the MM-GBSA calculations show that mutations of Glu361, His447, and Asn485 residues to alanine decrease the binding free energies of these systems by 35.8, 41.3, and 4.8 kJ mol^−1^, respectively, relative to the WT system. This indicates that Glu361 and His447 play important roles in substrate binding. On the basis of per-residue type, the free energy decomposition shows that the residues of Gly120, Glu361, His447, and Asn485 in the active site also have notable contributions to the substrate binding, in which the electrostatic and van der Waals interactions play predominant roles in binding the substrate in the active site.

The DHDFT results show that Glu361 and Gly120 play significant roles in the enzymatic catalysis. Glu361 serves as a base for abstracting the proton from the hydroxyl group of the substrate and facilitates the hydride transfer. The Gibbs free energy barrier for the hydride transfer is lowered by 22.6 kJ mol^−1^ upon inclusion of the Glu361 residue in our model. The hydrogen bond interaction between Gly120N–H and N5 of FAD also enhances the hydride transfer and lowers the barrier height of the hydride transfer further. We note that there is a good mechanistic agreement between the QM/MM MD simulations and our best DHDFT results.

In summary, our QM/MM MD simulations and high-level DHDFT using small model systems offer two complementary perspectives on the catalytic mechanism for the hydride transfer in ChOx. The combination of these two approaches gives an in-depth understanding of this flavoenzyme catalysis. More specifically, the QM/MM MD simulations show that the His447 and Glu361 residues play important roles in substrate binding. Our DHDFT models efficiently demonstrate the effects of the Glu361 and Gly120 residues on the reaction barrier height for the hydride transfer. The good agreement between the two approaches for the reaction barrier height of the hydride transfer increases our confidence in the results obtained from these models.

## Methods

### Preparation of the enzyme-substrate complex model

The initial coordinates used to build the model for the present study were based on the X-ray crystal structure of oxidized cholesterol oxidase (PDB ID: 4U2T)^4^. The steroid substrate dehydroepiandrosterone (DHA) was modeled in this structure in the proposed Michaelis state, based on the structure of the DHA/enzyme complex^20^ and knowledge of the interactions between amino acid side chains and the substrate from previous crystallographic and mutagenesis results^4–6,12,21^. Specifically the substrate hydroxyl group was positioned so as to make hydrogen bond contact to NE2 of His447 and the C3 hydrogen atom was positioned above the flavin N5 atom. The protonation states of the ionizable residues were determined at pH = 5.2 with the H++ program^22^ and the neighboring hydrogen bond networks. The pH value used here is taken from experiment^4^. The FAD cofactor and the substrate as well as the protein were described by the AMBER GAFF force field^23^ and the AMBER14SB force field^24^, respectively. The electrostatic potential (ESP) was calculated at the HF/6–31 G(d) level of theory using Gaussian 09 program suite^25^. The computed ESP of FAD and the substrate were fit using restrained electrostatic potential (RESP) charge^26^ fitting in *antechamber*. The whole system was solvated into an 88 × 84 × 103 Å rectangular box of TIP3P water^27^ with a 12 Å buffer distance between the box edge and the nearest solute atoms. The system was neutralized by adding Cl^–^ ions. The protons were added automatically, and the topology parameters as well as initial coordinates were generated by the *tleap* AmberTool. After the energy minimization, the system was heated gradually from 0 to 300 K for 100 ps, and another 100 ps MD simulation was carried out to relax the system density to about 1.0 g cm^–3^. Finally, the standard 10 ns MD simulation under isothermal-isobaric (NPT) ensemble (NTP = 1) was employed with an integration time step of 2 fs *via* using the periodic boundary condition. The Berendsen barostat was used for pressure-control. The cutoff value was set to 10 Å for van der Waals and electrostatic interaction calculations. Long-range electrostatic interactions were dealt with by the Particle Mesh Ewald (PME) method^28,29^. The Langevin thermostat (NTT = 3) was used to maintain the temperature at 300 K. All the bonds involving hydrogen atoms were constrained by using the SHAKE scheme^30^. The RMSD was used to estimate the stability of the backbone of the enzyme, and the last 2 ns trajectories were utilized for the protein-ligand interaction decomposition of the binding free energy for both the WT and mutant systems (Glu361Ala, His447Ala, and Asn485Ala). Hou *et al*. reported that the MM-GBSA method with the generalized Born model performs well for binding free energy estimations^31,32^. In the current study, the MMPBSA.py module^33^ was then applied for the post-processing in which 1000 snapshots from an ensemble of conformations are used to calculate the free energy. This method has been also used to analyze the binding free energy for protein-ligand interactions for the WT and mutant systems. The per-residue decomposition was applied to estimate the contribution from each residue to the total binding free energy. The whole MD simulations were accomplished by applying the AMBER 14 software^34^.

### QM/MM MD simulations

Development and applications of combined QM/MM methods for enzymes have been extensively reviewed in the past^35–37^. In order to obtain the barrier height of the hydride transfer, QM/MM MD simulations have been carried out for this enzyme, considering the influence from the surrounding residues to the reaction site. The residues: Lys225, Asn119, Gly120, Glu361, His447, Asn485, the FAD cofactor, and the substrate are considered as the QM region (103 atoms in total, see Fig. 2). The QM/MM boundary settings were determined according to the pseudo-bond rule^38^. These pseudo-bonds, C_ps_(*sp* ^*3*^)–C(*sp* ^*3*^), C_ps_(*sp* ^*3*^)–C(*sp* ^2^, carbonyl) and C_ps_(*sp* ^*3*^)–N(*sp* ^*3*^) with accurate parameterization are preferred to be used when cutting the QM and MM regions. The specified sets are listed below in detail: (1) Lys227: only the –NH3^+^ and the adjacent –CH_2_ groups included (C_ps_(*sp* ^*3*^)–C(*sp* ^*3*^) type); (2) Asn119: only the –C = O group forming peptide bond with –NH group of Gly120 included (C_ps_(*sp* ^*3*^)–C(*sp* ^2^, carbonyl) type); (3) Gly120: the whole Gly120 residue included and the –NH group of Gly121 included (C_ps_(*sp* ^*3*^)–N(*sp* ^*3*^) type); (4) Glu361: only the –COO^–^ and the adjacent –CH_2_ groups included (C_ps_(*sp* ^*3*^)–C(*sp* ^*3*^) type); (5) His447: the imidazole ring with one –CH_2_ group included (C_ps_(*sp* ^*3*^)–N(*sp* ^*3*^) type); (6) Asn485: the –NH_2_ and two adjacent –C = O and –CH_2_ groups included (C_ps_(*sp* ^*3*^)–C(*sp* ^*3*^) type); (7) FAD: without the long chain (C_ps_(*sp* ^*3*^)–C(*sp* ^*3*^) type); (8) DHA: only one six-membered ring with the –OH group was included as well as two adjacent short chains (C_ps_(*sp* ^*3*^)–C(*sp* ^2^, carbonyl) and C_ps_(*sp* ^*3*^)–C(*sp* ^*3*^) types). All the atoms in QM region are treated using the B3LYP/6–31 G(d) level of theory and atoms in the MM region are described by the AMBER99SB force field^39^. This approach has been successfully applied to other enzymatic reactions in the past decade^40,41^. The TIP3P model was used for the solvent water molecules^27^. The QM/MM boundary was addressed by the pseudo-bond approach^37,38^ with improved parameters^42^. The spherical boundary condition was applied, in which all atoms within a radius of 25 Å from the spherical center atom were allowed to freely move. The 12 and 18 Å cutoffs were used for van der Waals and electrostatic interactions among MM atoms, respectively. Following the interactive minimization, the minimum reaction energy path was mapped out by using the reaction coordinate driving method (RCD). Then, a 200 ps MD simulation was used to equilibrate the MM part with the QM subsystem constrained. The obtained snapshot was used for the subsequent QM/MM MD simulations combined with the umbrella sampling^43^. A total of 43 umbrella windows were employed to cover the reaction coordinate from –1.59 to 2.61 Å. For each window, 20 ps QM/MM MD simulations have been carried out with a time step of 1 fs by Beeman algorithm^44^. Berendsen thermostat method^45^ was used to control the system temperature at 300 K. The first 5 ps simulations were used for the equilibration and then another 15 ps simulations were used to obtain the PMF by the weighted histogram analysis method (WHAM)^46^. The appropriate step size and harmonic force constant for each window were chosen to guarantee the sufficient sampling. All the QM/MM MD simulations were carried out by the modified Q-Chem 4.0^47^ and Tinker 4.2 programs^48^.

### High-level double-hybrid DFT simulations

High-level DHDFT calculations using the B2GP-PLYP functional^49^ were performed in order to obtain accurate reaction energies and barrier heights of the hydride transfer from the C3 atom of the substrate to the N5 atom of the FAD cofactor. DHDFT methods include non-local electron correlation from second-order Møller–Plesset (MP2) perturbation theory in addition to the regular ingredients of hybrid DFT^50^. These methods overcome limitations of traditional DFT methods and display excellent performance for challenging chemical problems^50–54^. Due to the slow basis set convergence of the MP2-type correlation component^55^, the DHDFT calculations were carried out in conjunction with the quadruple-zeta Def2-QZVPP basis set^56^.

The B3LYP-D3/6–31+G(2df,p) level of theory was applied to fully optimize the geometries in our models **A**, **B**, and **C** (see Fig. 7). Based on these optimized geometries, single-point energies at RI-B2GP-PLYP/Def2-QZVPP level were carried out using the ORCA program^57^ (RI stands for resolution of identity approximation applied in the MP2 step)^58,59^. Zero-point vibrational energy (ZPVE), enthalpic, and entropic corrections have been obtained from the B3LYP-D3/6–31+G(2df,p) harmonic frequency calculations for converting the electronic RI-B2GP-PLYP/Def2-QZVPP reaction energies and barrier heights into Gibbs free reaction energies and barrier heights at 298 K. Empirical dispersion corrections were included using the Becke–Johnson damping potential^60^ as recommended by Grimme *et al*. (denoted by the suffix D3)^61–63^. The equilibrium structures in the unconstrained optimizations were verified to have all real harmonic frequencies and the transition structures were confirmed to have only one imaginary frequency. The connectivities of the transition structures were confirmed by performing intrinsic reaction coordinate (IRC) calculations^64,65^. The enzyme-like environment was simulated by a homogeneous polarizable continuum model with the dielectric constant of 4.0^66–68^ using the conductor-like polarizable continuum model (CPCM) at the HF/6–31+G(d) level of theory in conjunction with UAHF atomic radii, as recommended by Takano and Houk^69^.

All the above geometry optimization, harmonic frequency, and enzyme-like environment solvent correction calculations were performed using the Gaussian 09 program suite^25^.

## Electronic supplementary material

Supporting Information
