# Supplementary material for: Computational insights for the hydride transfer and distinctive roles of key residues in cholesterol oxidase
Source: Sci Rep. 2017 Dec 8;7:17265. doi: 10.1038/s41598-017-17503-x (PMC5722936; doi:10.1038/s41598-017-17503-x)
Supplement: Supplementary file 1 — Supporting Information [file 41598_2017_17503_MOESM1_ESM.pdf]

Supplementary Information

Computational insights for the hydride transfer and distinctive roles of key residues in cholesterol oxidase

Li-Juan Yu,[†] Emily Golden,[†] Nanhao Chen,[§,¶] Yuan Zhao,[*,‡] Alice Vrielink,[†] and Amir Karton[*,†]

[†]School of Molecular Sciences, The University of Western Australia, Perth, WA 6009, Australia.

[‡]The Key Laboratory of Natural Medicine and Immuno-Engineering, Henan University, Kaifeng 475004, China.

[§]School of Pharmaceutical Sciences, Sun Yat-sen University, Guangzhou 510006, China.

[¶]Department of Chemistry, University of California, Davis, California 95616, United States

[*]Corresponding Authors E-mail addresses: zhaoyuan@henu.edu.cn (Y. Zhao) and amir.karton@uwa.edu.au (A. Karton).

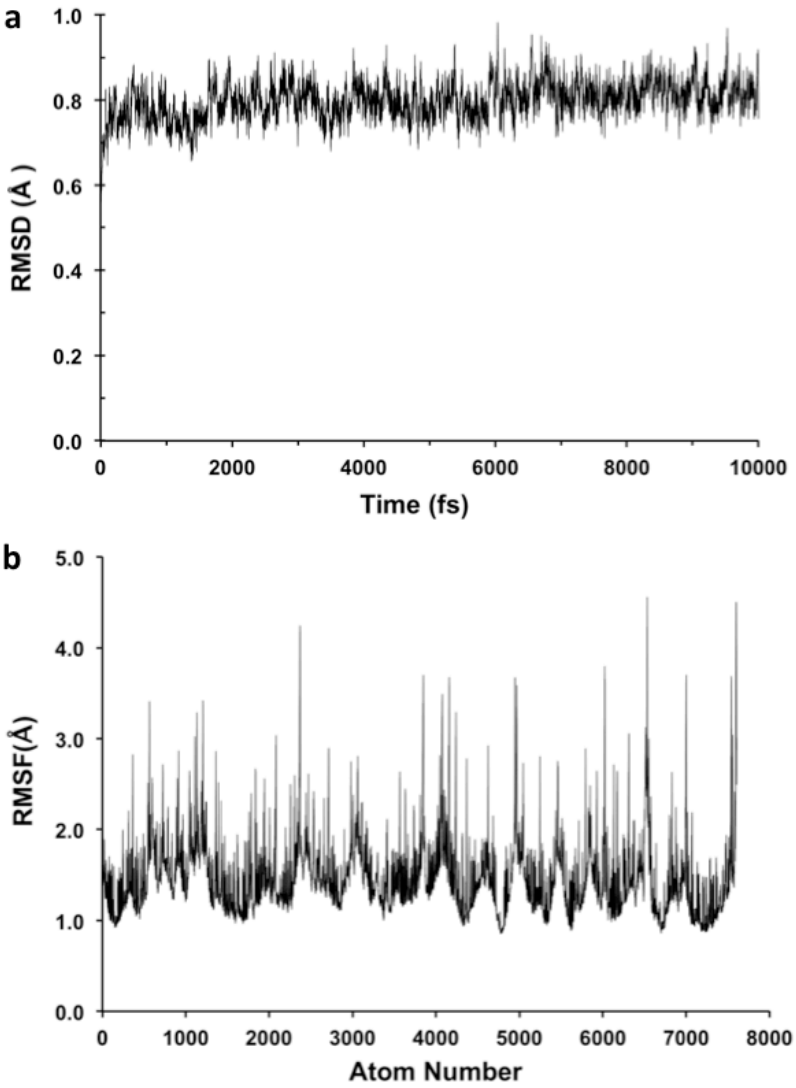

Supplementary Figure S1

(**a**) The root-mean-square deviation (RMSD) results of the protein backbone and (**b**) the root-mean-square fluctuation (RMSF) results of atoms.

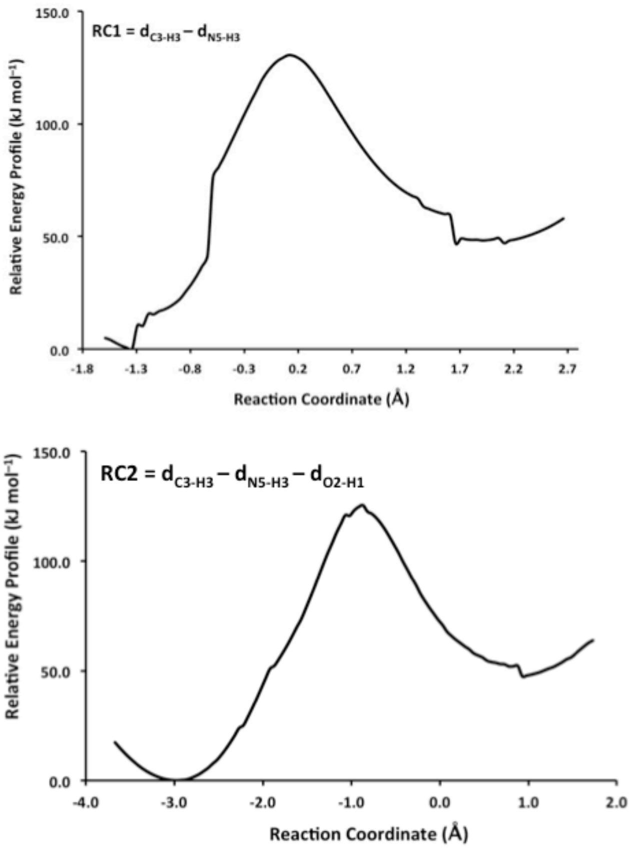

Supplementary Figure S2

Relative energy profiles of the hydride transfer as well as the proton transfer for ChOx by using QM/MM scan with different reaction coordinates.

a

b

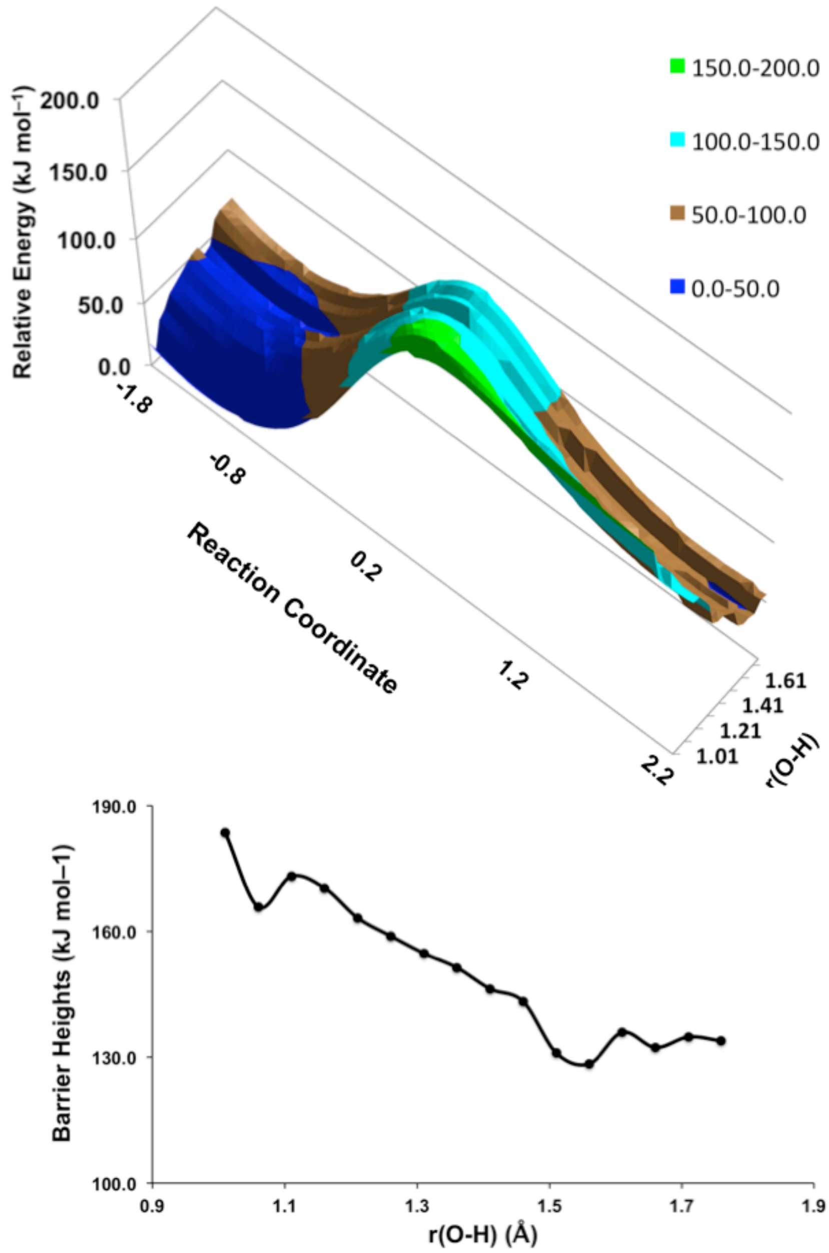

Supplementary Figure S3

(**a**) Relative energy profiles of the hydride transfer as well as the proton transfer for ChOx by using 2D scan. Here, the O1–H1 distance was fixed at 1.01, 1.06, 1.11, 1.16, 1.21, 1.26, 1.31, 1.36, 1.41, 1.46, 1.51, 1.56, 1.61, 1.66, 1.71, and 1.76 Å separately. (**b**) Summary of the hydride transfer barrier height for each scan.

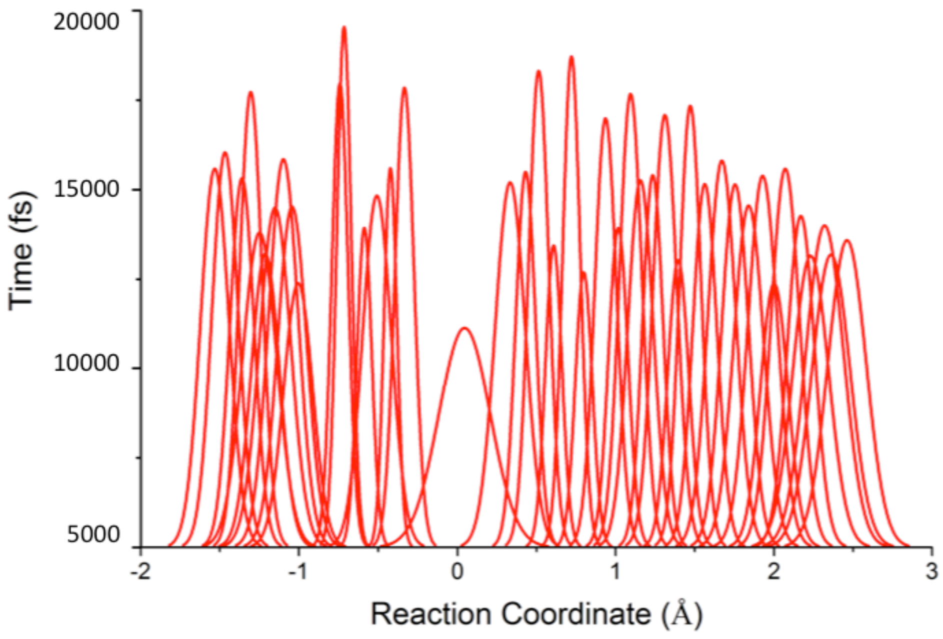

Supplementary Figure S4

Configurational overlap for the current 1D reaction coordinate sampling in the range of 5ps to 20ps.

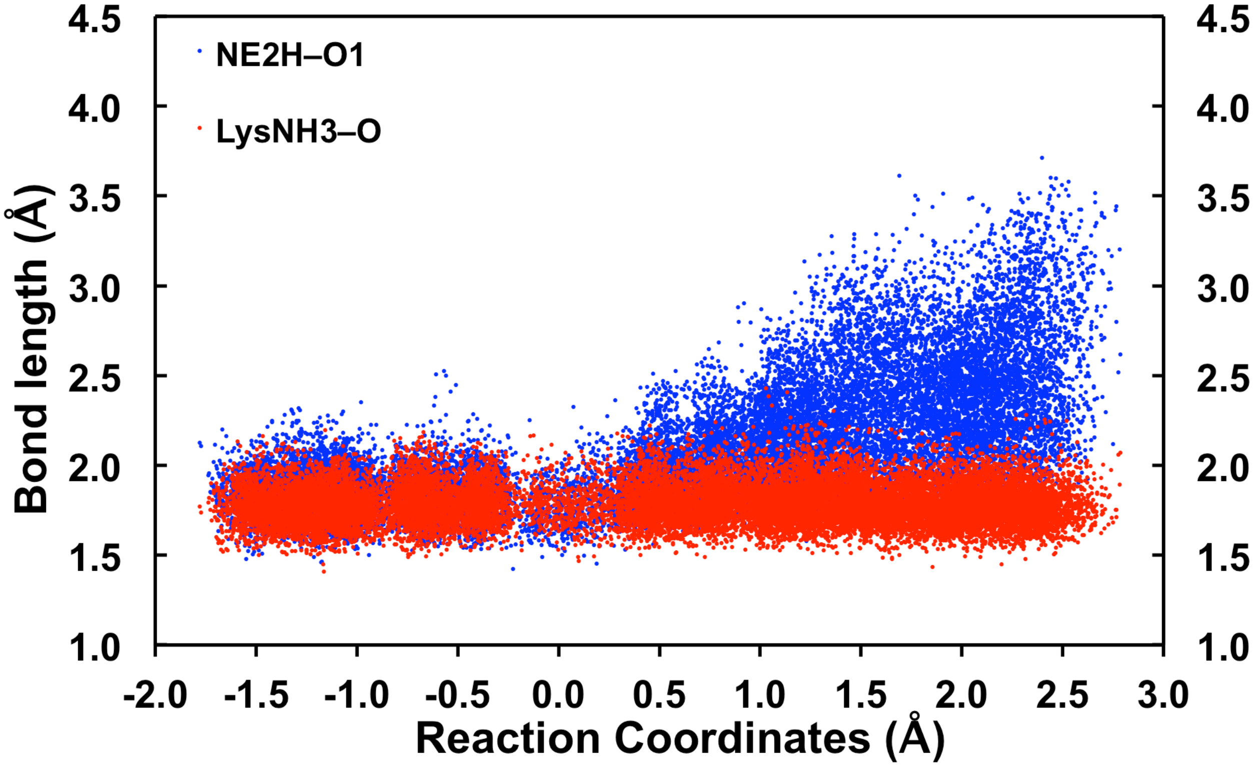

Supplementary Figure S5

Statistics of NE2H–O1 and $LysNH_3$–O bond lengths from snapshots along RC1.

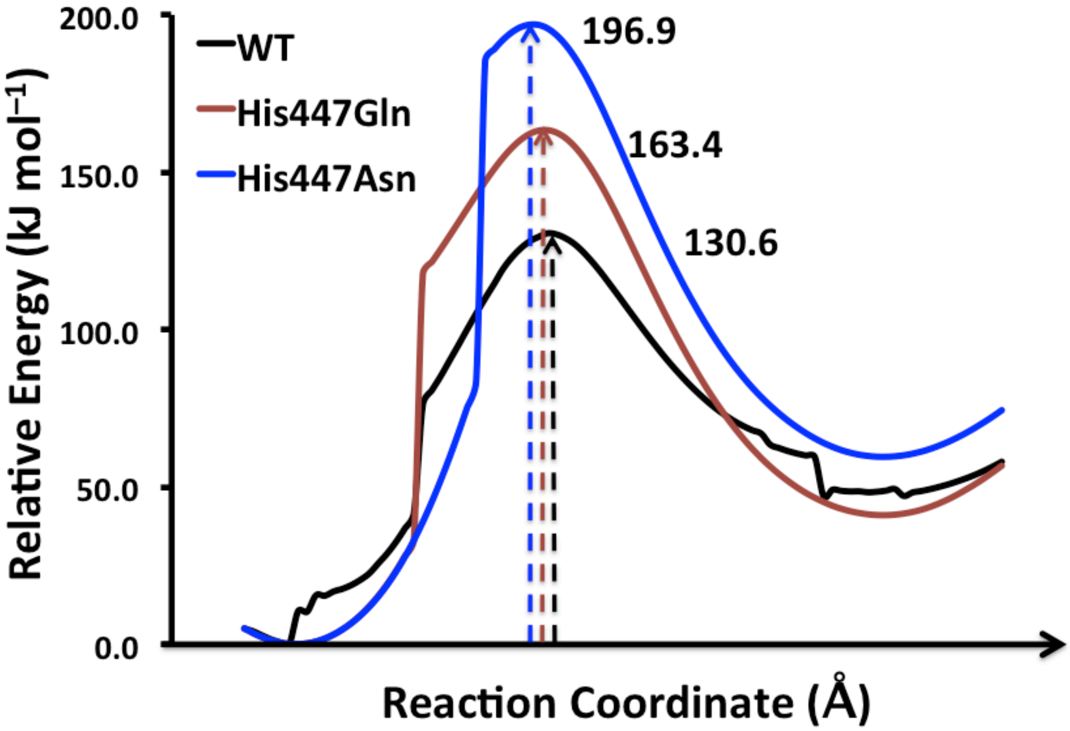

Supplementary Figure S6

The predicted relative energies for the hydride transfer in His447Gln (dark red), His447Asn (blue), and the wild-type (black) cholesterol oxidase.

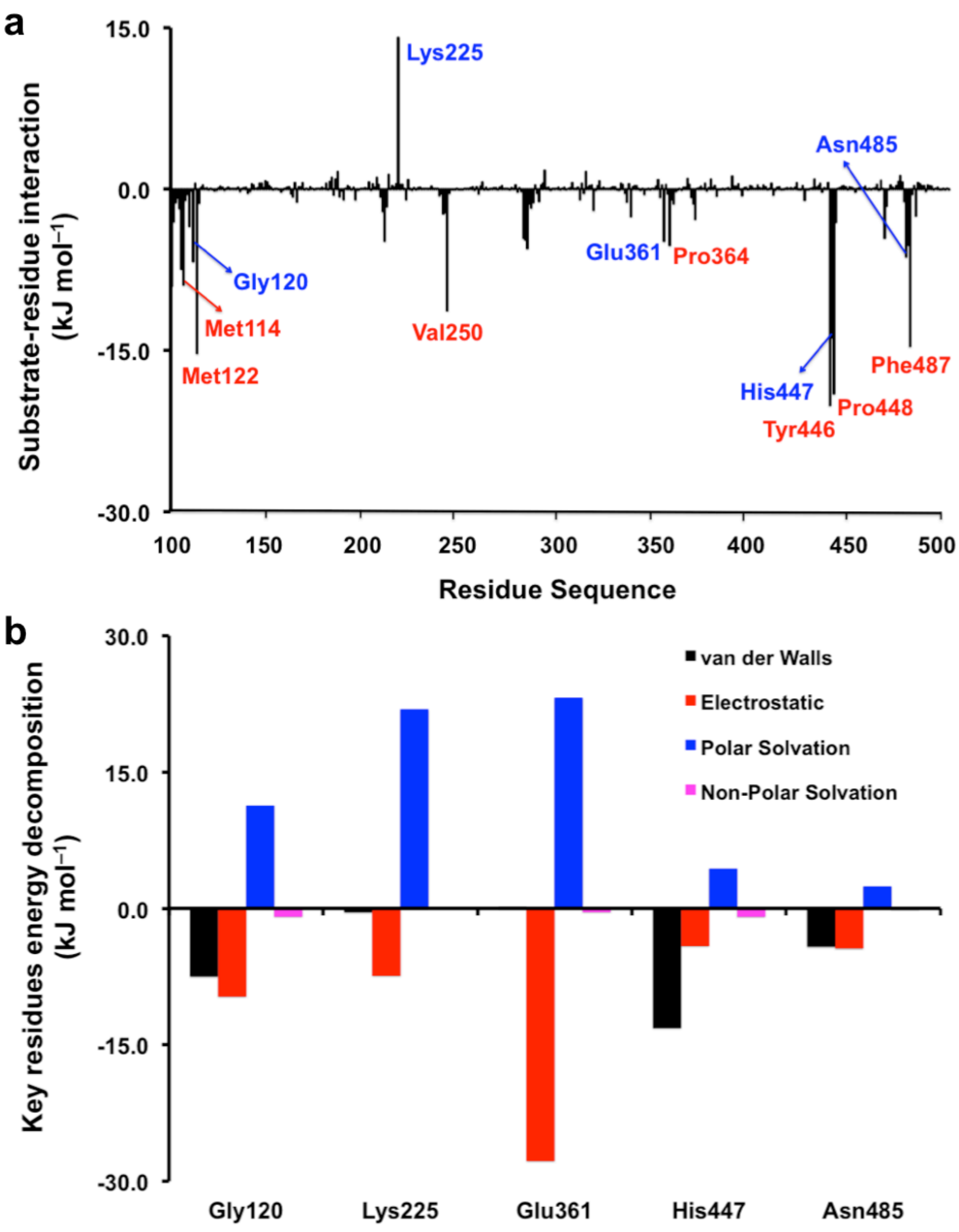

Supplementary Figure S7

Free energy decomposition based on the per-residue type (**a**) and Free energy decomposition for the key residues (**b**) in ChOx.

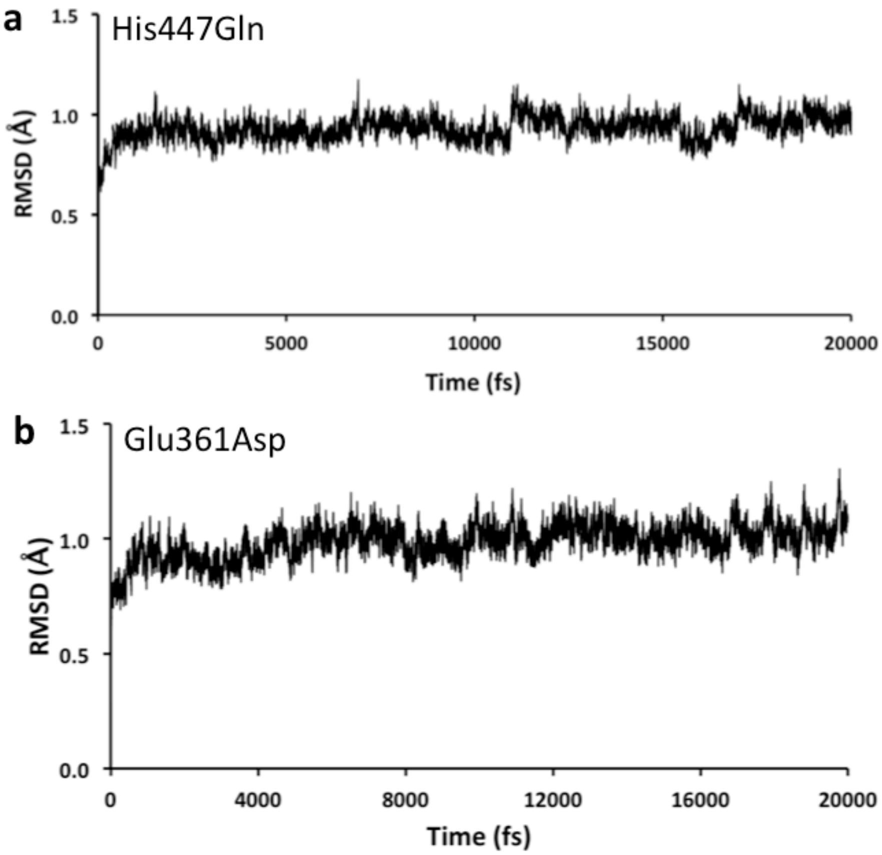

Supplementary Figure S8

(a) The root-mean-square deviation (RMSD) of the protein backbone for the His447Gln mutant; (**b**) The RMSD of the backbone for the Glu361Asp mutant.

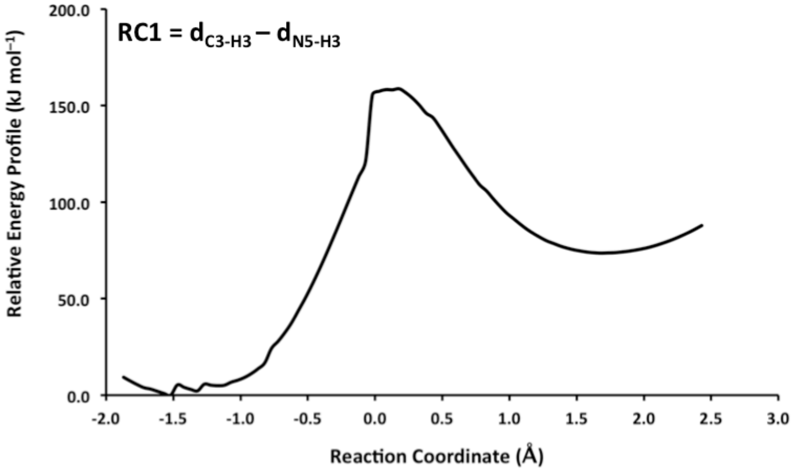

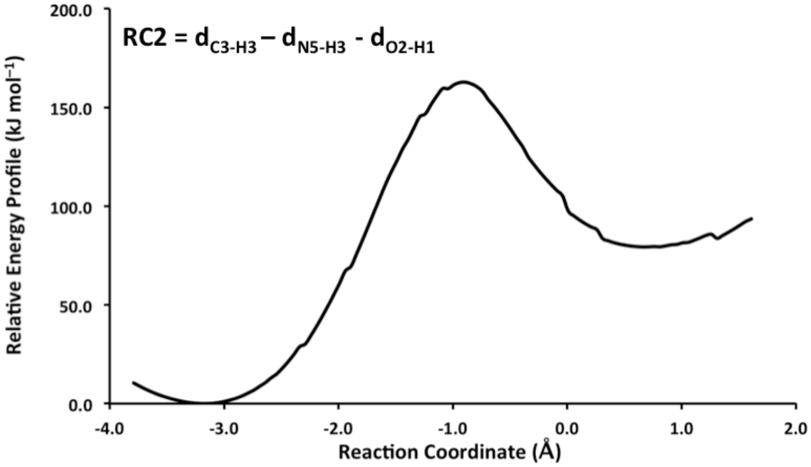

Supplementary Figure S9

Relative energy profiles of the hydride transfer as well as the proton transfer for His447Gln mutant by using QM/MM scan with different reaction coordinates.

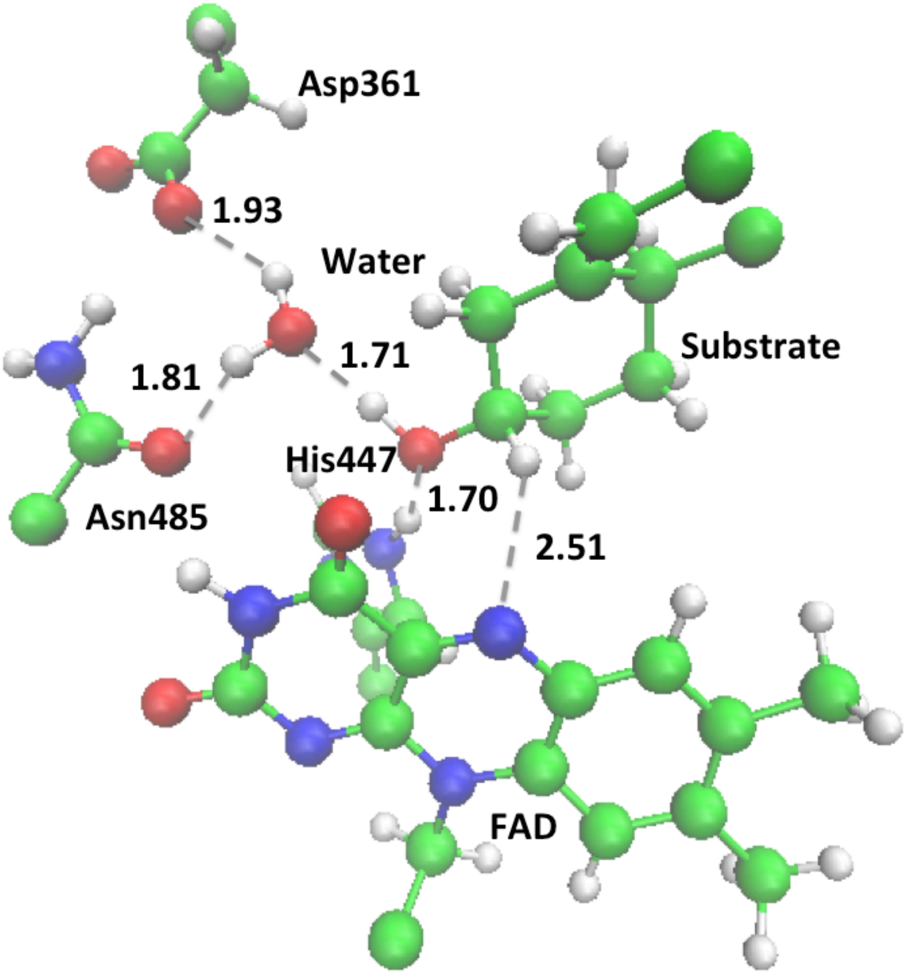

Supplementary Figure S10

Overall view of QM/MM model for Glu361Asp mutant with active site. In order to clearly show the water molecule and the hydrogen bond networking, only FAD, the substrate, Asp361, Water, His447, and Asn485 in the QM region are displayed. The Lys225, Asn119, and Gly120 in the other side of FAD are not displayed in this figure. Hydrogen bonds are shown as grey dashed lines (in Å).

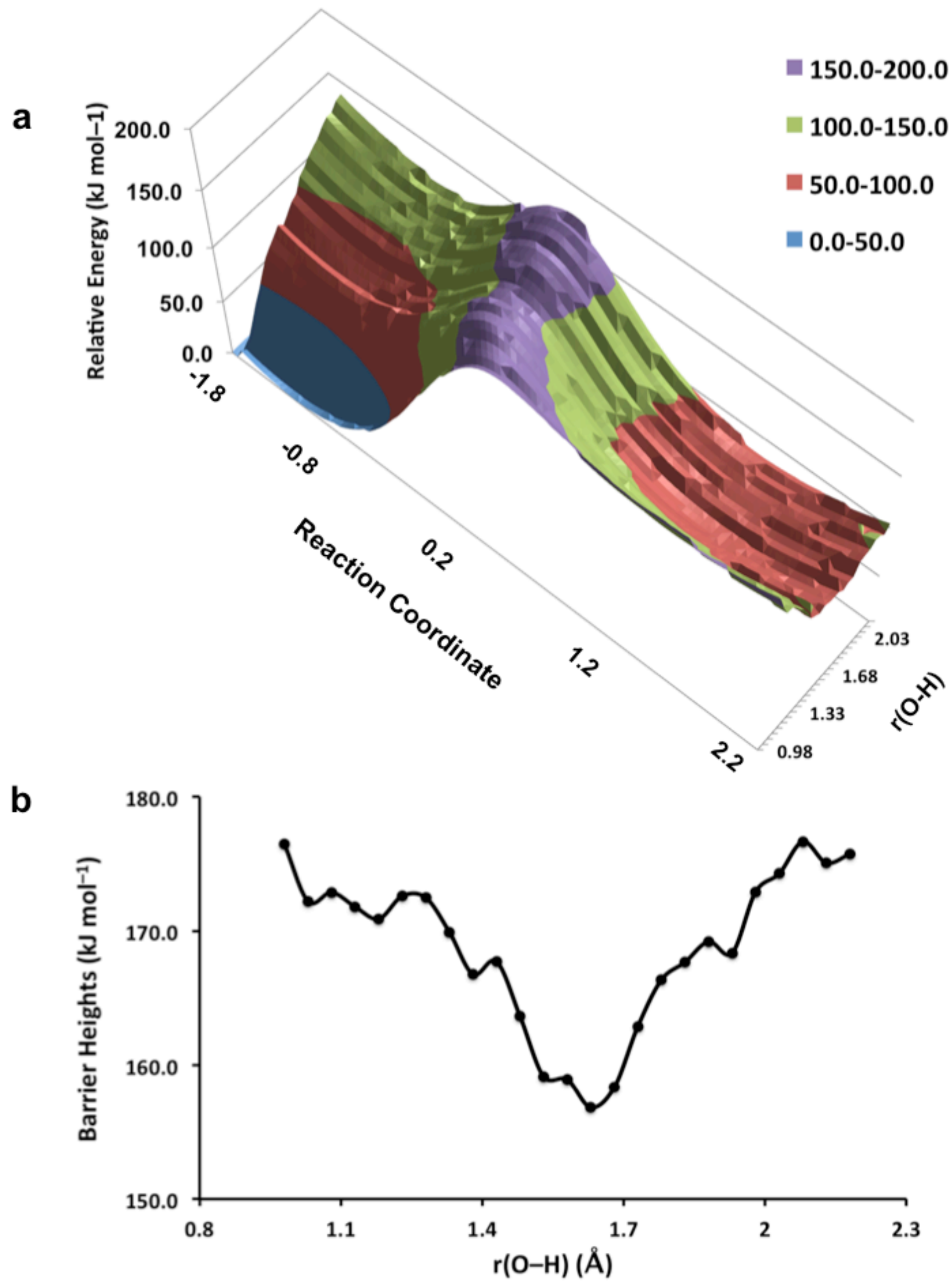

Supplementary Figure S11

(**a**) Relative energy profiles of the hydride transfer as well as the proton transfer for His447Gln mutant by using 2D scan. Here, the O1–H1 distance was fixed at 0.98, 1.03, 1.08, 1.13, 1.18, 1.23, 1.28, 1.33, 1.38, 1.43, 1.48, 1.53, 1.58, 1.63, 1.68, 1.73, 1.78, 1.83, 1.88, 1.93, 1.98, 2.03, 2.08, 2.13, and 2.18 Å separately. (**b**) Simplified summary for the barrier heights for each separate scan.

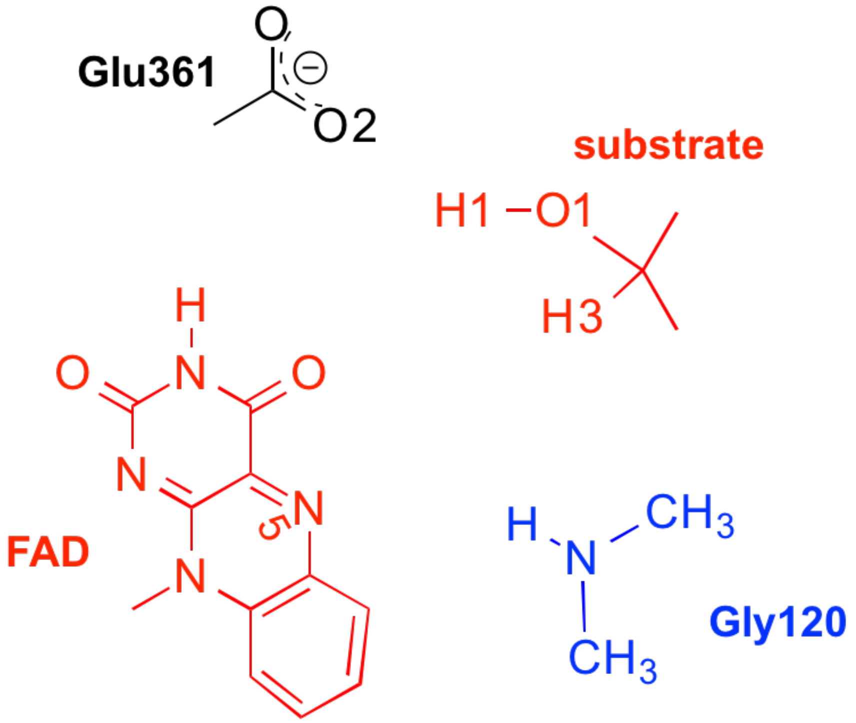

Supplementary Figure S12

Atoms included in DHDFT models **A**, **B**, and **C**.

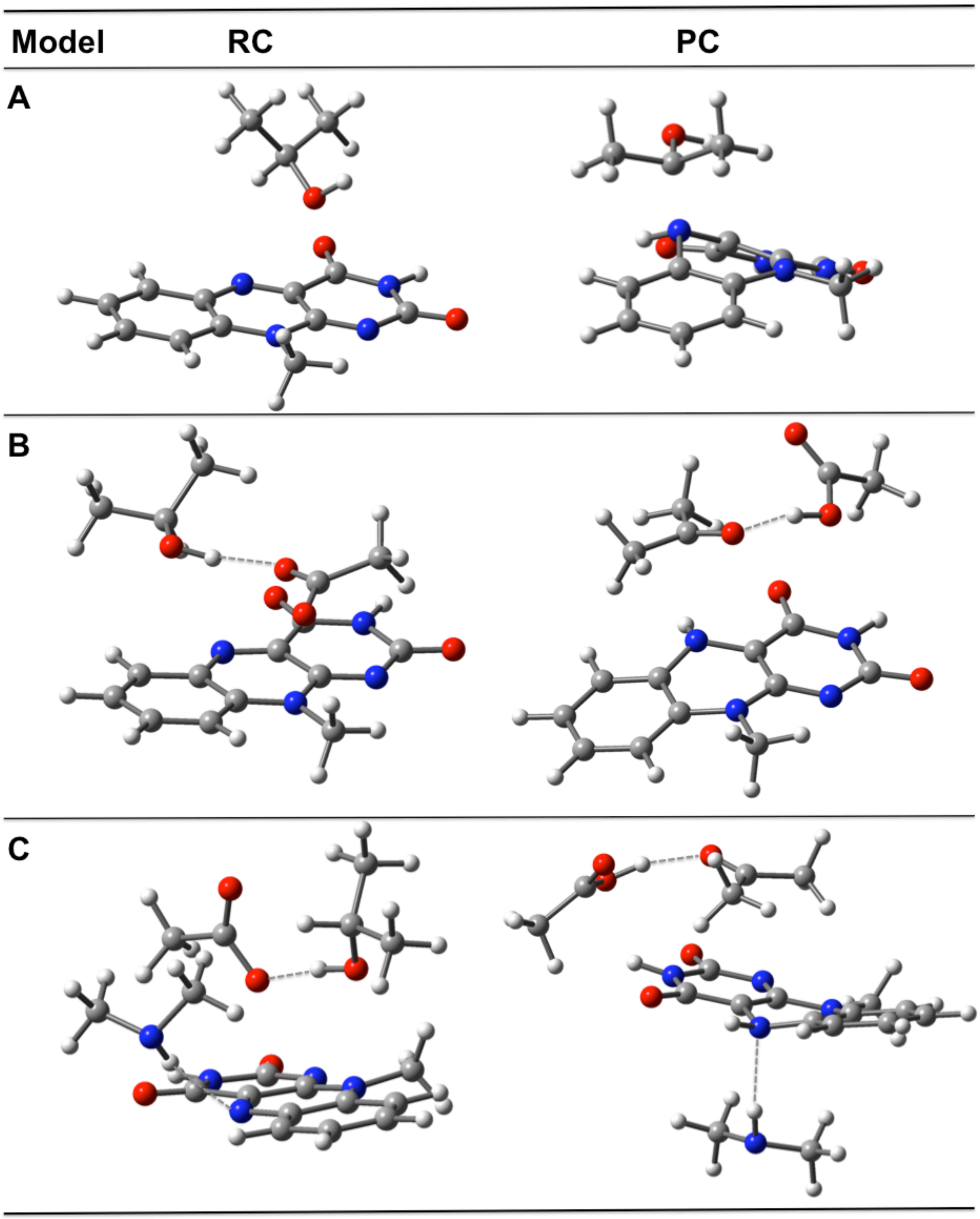

Supplementary Figure S13

B3LYP-D3/6-31+G(2df,p) optimised reactant complexes (RCs) and product complexes (PCs) located on the potential energy profiles in models **A**, **B**, and **C**. Hydrogen bonds are shown as grey dashed lines. Atomic color scheme: H, white; C, grey; N, blue; O, red.

Supplementary Table S1. Barrier heights (BH) and reaction energies (RE) (in kJ mol^{-1}) calculated at the RI-B2GP-PLYP/Def2-QZVPP//B3LYP-D3/6-31+G(2df,p) level of theory.

**Models**	**Energies**	**Δ*G*298 (kJ/mol)**		**Δ*H*298 (kJ/mol)**	
		**Gas**	**CPCM(*ε* = 4.0)**	**Gas**	**CPCM(*ε* = 4.0)**
**A**	BH	175.8	136.5	157.1	117.8
	RE	79.5	37.1	53.5	11.1
**B**	BH	96.3	113.9	85.4	103.0
	RE	–13.6	2.2	–26.0	–10.2
**C**	BH	86.5	102.1	84.8	100.4
	RE	–24.4	–14.7	–30.5	–20.8

Supplementary Table S2. Selected distances (Å) of the optimized structures for the reactants complexes (RCs), transition structures (TSs), and products complexes (PCs) from the reactions listed in Figure 5 and Figure S6. The distance labels are shown in Scheme 1, R_1 = O2•••H1 and R_2 = O1•••H1.

Model	Distance	RC	TS	PC
**B**	R_1	1.715	1.046	0.994
	R_2	0.992	1.465	1.702
**C**	R_1	1.666	1.042	0.993
	R_2	1.000	1.475	1.718

Supplementary Table S3. B3LYP-D3/6-31+G(2df,p) optimized geometries (Å) for all structures in models **A**–**C**.

Model **A**

React

C	1.156941	-2.568918	-1.607534
C	4.342069	-0.192734	-0.103878
C	2.835385	0.931390	1.394006
C	1.771118	0.154296	0.905242
C	-0.461893	-0.337831	0.950953
N	0.516686	0.365324	1.413495
C	-1.844956	-0.061810	1.453137
C	-2.588174	-1.888338	-0.079704
C	-0.328133	-1.370603	-0.073067
C	2.008886	-0.813703	-0.104419
N	0.942736	-1.568130	-0.560261
C	-0.109063	3.464503	-1.700817
C	-0.950399	2.570392	-0.793688
O	-1.004534	1.231802	-1.317690
C	-2.353327	3.117710	-0.553224
H	-0.445033	2.455018	0.170447
H	-2.911245	2.462765	0.120321
H	-2.305511	4.112446	-0.098695
H	-2.901975	3.208922	-1.500137
H	-0.592592	3.593344	-2.677322
H	0.019624	4.456802	-1.255420
H	0.879523	3.024288	-1.861003
H	1.530914	-2.081707	-2.512155
H	1.881889	-3.312426	-1.266292
H	0.202421	-3.046090	-1.811377
H	-3.744949	-0.768916	1.206634
H	4.932928	1.364243	1.273253
O	-2.120610	0.815171	2.245463
N	-2.785634	-0.903496	0.911919
N	-1.307126	-2.092651	-0.539407
O	-3.551665	-2.507027	-0.484930
H	2.606233	1.657214	2.165488
H	5.342718	-0.328387	-0.499768
H	3.520736	-1.702938	-1.374429
C	4.113316	0.763813	0.896627
C	3.311546	-0.975271	-0.603186
H	-1.587732	1.221142	-2.085211

TS

C	-0.308187	-3.173648	0.940592
C	-3.972374	-1.367280	-0.230615
C	-2.791127	0.543342	-1.077756
C	-1.577982	-0.024052	-0.686482
C	0.795621	0.043505	-0.629585
N	-0.384988	0.666119	-0.924687
C	2.023746	0.727251	-0.976840
C	3.192558	-1.227475	0.031190
C	0.868240	-1.217175	0.012110
C	-1.549506	-1.293156	-0.064505

N	-0.333179	-1.857020	0.313036
C	-1.565792	2.223046	1.599984
C	-0.254220	2.475596	0.936037
O	0.820331	1.827136	1.415502
C	0.077628	3.809246	0.346585
H	-0.404696	1.696276	-0.648775
H	1.020667	3.759807	-0.199341
H	-0.712721	4.144098	-0.331024
H	0.164332	4.562414	1.147986
H	-1.583205	2.688428	2.598932
H	-2.388707	2.646843	1.020806
H	-1.762869	1.151205	1.726228
H	-0.888331	-3.162440	1.869134
H	-0.727852	-3.927765	0.266131
H	0.729727	-3.416556	1.154448
H	4.048635	0.448127	-0.820124
H	-4.927729	0.333592	-1.155951
O	2.063671	1.841032	-1.494992
N	3.153193	0.018512	-0.628513
N	1.989822	-1.828060	0.321809
O	4.275370	-1.711284	0.311592
H	-2.769834	1.509827	-1.570524
H	-4.899702	-1.898490	-0.047796
H	-2.782604	-2.923079	0.627479
C	-3.991041	-0.116231	-0.848825
C	-2.770239	-1.952134	0.153049
H	0.572034	1.030765	1.904432

Prod

C	0.071131	-2.919096	1.281256
C	-3.503780	-1.856505	-0.786300
C	-2.610047	0.316724	-1.265715
C	-1.480845	0.000853	-0.526110
C	0.866337	0.387664	-0.239253
N	-0.445810	1.008036	-0.293577
C	1.882648	0.986501	-1.040195
C	3.322496	-0.851409	-0.167348
C	1.050101	-0.897457	0.271309
C	-1.294355	-1.274445	0.044759
N	-0.092266	-1.609827	0.659992
C	-0.861171	1.119741	2.217836
C	-0.726023	1.972294	0.965810
O	0.352771	2.833209	1.016140
C	-1.958392	2.806294	0.666138
H	-0.364562	1.664959	-1.088639
H	-1.837837	3.351466	-0.274370
H	-2.859771	2.194436	0.628705
H	-2.067808	3.541769	1.466121
H	-1.015986	1.796781	3.060838
H	-1.711894	0.438727	2.156459
H	0.047700	0.542903	2.399854
H	-0.709530	-3.065619	2.032473
H	0.024132	-3.727569	0.542133
H	1.053242	-2.944920	1.747691
H	3.883980	0.701143	-1.424632
H	-4.519071	-0.370061	-1.980482
O	1.699393	1.982967	-1.750917

N	3.085969	0.321390	-0.933180
N	2.221437	-1.489214	0.359096
O	4.466340	-1.254665	-0.056447
H	-2.690070	1.295537	-1.724978
H	-4.296662	-2.591167	-0.873349
H	-2.259593	-3.176739	0.349608
C	-3.634879	-0.614699	-1.405098
C	-2.352446	-2.189436	-0.080211
H	1.165755	2.321431	1.144774

Model **B**

React

C	1.508921	-2.907266	0.138256
C	-2.616052	-2.904508	-0.813131
C	-2.444016	-0.693778	-1.735639
C	-1.082177	-0.658275	-1.383753
C	0.859852	0.530431	-1.242152
N	-0.373255	0.479256	-1.632999
C	1.619981	1.799178	-1.441271
C	3.569632	0.585557	-0.448171
C	1.576408	-0.580258	-0.628770
C	-0.486207	-1.779535	-0.751104
N	0.860477	-1.734227	-0.459280
C	-3.905446	2.526857	0.072695
C	-2.526683	2.099137	0.569224
O	-2.656119	0.798335	1.108461
C	-1.959678	3.079918	1.601132
H	-1.837865	2.079920	-0.290921
H	-1.745710	0.485681	1.348182
C	1.617771	-0.196065	3.248962
C	0.383757	-0.580518	2.416752
O	-0.047435	-1.750064	2.509474
O	-0.064818	0.327931	1.650828
H	-0.988874	2.719127	1.952318
H	-1.829467	4.077999	1.163178
H	-2.637354	3.156413	2.460929
H	-4.614357	2.555117	0.909441
H	-3.866103	3.520512	-0.388431
H	-4.283858	1.812362	-0.666374
H	1.541793	0.837387	3.600305
H	1.751110	-0.878348	4.092762
H	2.502513	-0.262238	2.603606
H	1.076288	-3.070412	1.128297
H	1.352388	-3.770735	-0.514620
H	2.569180	-2.688256	0.225250
H	3.488662	2.543054	-1.090143
H	-4.261979	-1.825842	-1.711486
O	1.167768	2.812267	-1.940726
N	2.919203	1.712101	-0.998985
N	2.843656	-0.559857	-0.286149
O	4.750109	0.694203	-0.152801
H	-2.859184	0.195579	-2.193326
H	-3.222565	-3.767754	-0.558941
H	-0.862389	-3.735695	0.084128
C	-3.207008	-1.807556	-1.463035
C	-1.278862	-2.901011	-0.459020

TS

C	-2.229751	2.162402	-1.954337
C	-4.905451	-0.815983	-0.531988
C	-3.308986	-1.460199	1.143307
C	-2.419968	-0.478822	0.701669
C	-0.416714	0.683915	0.971647
N	-1.164709	-0.387751	1.319626
C	0.818006	0.929934	1.713182
C	1.175888	2.867459	0.184266
C	-0.719469	1.567535	-0.107992
C	-2.786350	0.359844	-0.376684
N	-1.901507	1.339762	-0.801362
C	-0.654901	-2.662410	-1.041687
C	0.312298	-1.907633	-0.126377
O	0.848606	-0.836736	-0.638039
C	1.206447	-2.793891	0.748050
H	-0.493751	-1.422727	0.789353
H	2.279808	-0.561997	-0.485550
C	5.577022	-0.643310	-0.627347
C	4.149738	-1.129390	-0.817099
O	3.885707	-2.212211	-1.314098
O	3.273206	-0.253797	-0.378361
H	1.712825	-2.180796	1.496859
H	0.619009	-3.563049	1.261828
H	1.961741	-3.277784	0.115769
H	-0.077044	-3.195534	-1.808868
H	-1.257013	-3.392271	-0.489815
H	-1.325425	-1.960830	-1.546189
H	5.777557	-0.511832	0.441143
H	6.278212	-1.363816	-1.050309
H	5.703659	0.334314	-1.102553
H	-2.421852	1.526246	-2.825208
H	-3.117960	2.775491	-1.757781
H	-1.379346	2.812771	-2.144854
H	2.423041	2.195577	1.673921
H	-5.221779	-2.411959	0.887837
O	1.233842	0.254085	2.647279
N	1.517939	2.032334	1.255516
N	-0.000579	2.608003	-0.469355
O	1.921223	3.796566	-0.105436
H	-2.986881	-2.084531	1.969847
H	-5.866162	-0.942362	-1.020981
H	-4.337202	0.800682	-1.814686
C	-4.546210	-1.639714	0.536129
C	-4.039952	0.176046	-0.983322

Prod

C	-2.385569	2.366033	-1.661401
C	-4.965739	-0.478530	0.179568
C	-3.099569	-1.380927	1.402245
C	-2.232219	-0.462056	0.811299
C	-0.110265	0.667639	0.736736
N	-0.868710	-0.498013	1.027290
C	1.171885	0.819180	1.302446
C	1.306845	2.948659	0.013555
C	-0.588676	1.628402	-0.139183

C	-2.744288	0.496760	-0.108108
N	-1.870073	1.420469	-0.694858
C	-1.097918	-2.923099	-1.338421
C	0.269660	-2.382834	-1.001532
O	0.824753	-1.597244	-1.755992
C	0.953786	-2.912621	0.235804
H	-0.542605	-0.974068	1.858898
H	2.327189	-0.891537	-1.378759
C	4.828230	-0.253919	0.543664
C	3.952859	-1.082251	-0.362283
O	4.030244	-2.292231	-0.467657
O	3.058098	-0.333885	-1.000206
H	1.374023	-2.088710	0.818440
H	0.267464	-3.492477	0.858115
H	1.793694	-3.543423	-0.076482
H	-1.041383	-4.015325	-1.432961
H	-1.802475	-2.703158	-0.530325
H	-1.460267	-2.486142	-2.270091
H	4.239200	-0.053888	1.447251
H	5.731407	-0.806067	0.806131
H	5.073927	0.704780	0.080135
H	-2.847527	1.830823	-2.500064
H	-3.140003	3.033610	-1.217180
H	-1.550766	2.969997	-2.010645
H	2.735073	2.151636	1.262507
H	-5.114339	-2.132275	1.551320
O	1.725621	0.000141	2.072899
N	1.797293	1.997261	0.923541
N	0.064342	2.728245	-0.504266
O	2.011639	3.925353	-0.254357
H	-2.690622	-2.095412	2.111474
H	-6.018747	-0.474921	-0.081460
H	-4.519082	1.192339	-1.094874
C	-4.463531	-1.399608	1.085225
C	-4.108319	0.469407	-0.403093

Model **C**

React

C	-0.685301	-3.043261	1.291448
C	-3.573286	-1.736717	-1.523585
C	-1.909974	-0.431458	-2.669142
C	-0.935527	-0.886862	-1.764496
C	1.230800	-0.816991	-1.039281
N	0.345141	-0.415914	-1.888089
C	2.612446	-0.261289	-1.127968
C	3.196310	-1.873507	0.684574
C	0.987110	-1.808285	0.004369
C	-1.296290	-1.777912	-0.724612
N	-0.313402	-2.216188	0.142500
C	-3.912364	0.593870	1.692827
C	-2.485754	1.017560	2.038974
O	-1.628200	-0.069140	1.740912
C	-2.344728	1.438377	3.503736
H	-2.207376	1.877818	1.414433
H	-0.711433	0.284136	1.554855
C	2.709339	2.140056	1.374030

C	1.219361	1.828104	1.563600
O	0.500067	2.650208	2.154162
O	0.824479	0.714050	1.074654
H	-1.317275	1.763743	3.684823
H	-3.019899	2.271324	3.741074
H	-2.584647	0.593684	4.162601
H	-4.197725	-0.286383	2.283679
H	-4.625050	1.398641	1.908624
H	-3.987604	0.331590	0.633036
H	2.970316	3.084304	1.856654
H	3.314438	1.329893	1.795482
H	2.940850	2.200540	0.305167
H	-1.411030	-2.483529	1.885855
H	-1.104810	-3.994161	0.949031
H	0.216087	-3.227830	1.868949
H	4.435792	-0.521952	-0.252972
H	-3.975050	-0.491535	-3.245741
O	2.950974	0.621095	-1.893825
N	3.487162	-0.872163	-0.265380
N	1.907671	-2.329403	0.771587
O	4.111635	-2.294710	1.373350
H	-1.591431	0.276257	-3.426062
H	-4.604355	-2.058077	-1.417772
H	-2.937895	-2.845224	0.189692
C	-3.221036	-0.852493	-2.555309
C	-2.632319	-2.194543	-0.616438
N	-0.101229	2.699416	-2.272906
H	0.301270	1.766844	-2.223009
C	0.975899	3.674478	-2.163961
H	1.756968	3.439729	-2.893229
H	0.589417	4.679895	-2.381758
H	1.439024	3.705403	-1.162048
C	-1.089954	2.853685	-1.207541
H	-1.627858	3.801849	-1.345399
H	-1.822370	2.042844	-1.265185
H	-0.655788	2.851062	-0.196126

TS

C	-1.786518	-3.674828	0.201114
C	-4.715081	-0.821118	1.371809
C	-3.300867	0.963366	0.599712
C	-2.337437	0.037177	0.196042
C	-0.319885	-0.409121	-0.889893
N	-1.136840	0.518054	-0.344455
C	0.831255	0.064934	-1.646929
C	1.433528	-2.334126	-1.937081
C	-0.480491	-1.817757	-0.738487
C	-2.565601	-1.346251	0.375007
N	-1.600096	-2.253621	-0.043328
C	-0.463700	0.510238	2.907061
C	0.425541	0.733359	1.681718
O	1.066958	-0.313557	1.250674
C	1.159579	2.078450	1.643807
H	-0.462717	0.932677	0.742443
H	2.485427	-0.226418	0.854051
C	5.759789	0.154810	0.793184
C	4.352254	0.183933	1.363862

O	4.108370	0.571711	2.494323
O	3.463329	-0.245246	0.494108
H	1.618676	2.219239	0.662666
H	0.471517	2.908906	1.834872
H	1.950664	2.078741	2.404028
H	0.172656	0.412431	3.796945
H	-1.158431	1.341904	3.064448
H	-1.037546	-0.414154	2.794792
H	6.478898	0.463106	1.553007
H	5.994972	-0.851626	0.433516
H	5.817134	0.828004	-0.068587
H	-1.933016	-3.853756	1.271691
H	-2.657328	-4.055542	-0.346587
H	-0.892242	-4.186924	-0.146213
H	2.487731	-0.688025	-2.580388
H	-5.227767	1.272228	1.503485
O	1.099230	1.245705	-1.853488
N	1.624563	-0.956561	-2.128880
N	0.318363	-2.731385	-1.245164
O	2.249366	-3.114177	-2.412940
H	-3.075864	2.014305	0.445851
H	-5.635977	-1.164395	1.832495
H	-3.966669	-2.811763	1.122304
C	-4.488004	0.544290	1.188556
C	-3.769581	-1.759545	0.968025
N	-1.299215	3.505583	-1.147785
H	-0.924734	2.587789	-0.912117
C	-2.070970	3.356625	-2.371260
H	-2.819106	2.568314	-2.237809
H	-2.598946	4.292506	-2.601129
H	-1.451255	3.093377	-3.249355
C	-0.193916	4.439191	-1.303619
H	-0.582347	5.452593	-1.476186
H	0.403358	4.455783	-0.387200
H	0.478102	4.178945	-2.140932

Prod

C	2.840398	0.612113	2.274377
C	4.168828	-2.602026	-0.182061
C	2.394049	-2.145993	-1.743658
C	1.936598	-1.091439	-0.955833
C	0.193844	0.432761	-0.326530
N	0.884582	-0.279048	-1.344154
C	-1.117545	0.888838	-0.570821
C	-0.966230	2.011768	1.645992
C	0.825870	0.768196	0.860330
C	2.597250	-0.790739	0.267464
N	2.129187	0.269462	1.060944
C	-0.227986	-2.032131	2.168750
C	-1.451031	-1.852710	1.298357
O	-2.437904	-1.293868	1.755938
C	-1.457941	-2.449239	-0.088622
H	0.266822	-0.618923	-2.070435
H	-3.745457	-0.643909	0.851023
C	-5.084906	0.353156	-1.894738
C	-4.619966	-0.636340	-0.858282
O	-4.523484	-1.832430	-1.045758

O	-4.297219	-0.037147	0.290457
H	-1.698028	-1.669348	-0.818667
H	-0.502426	-2.912451	-0.341486
H	-2.268788	-3.181527	-0.153615
H	-0.369898	-2.948763	2.757947
H	0.684213	-2.147354	1.582412
H	-0.139243	-1.186809	2.854068
H	-5.601222	-0.163652	-2.704401
H	-5.727737	1.115909	-1.447057
H	-4.183581	0.845566	-2.277310
H	2.831522	-0.214023	3.001556
H	3.883207	0.856562	2.039373
H	2.340623	1.469642	2.719991
H	-2.598740	1.941292	0.383969
H	3.857515	-3.703712	-2.006325
O	-1.781219	0.618921	-1.596886
N	-1.627663	1.670661	0.453226
N	0.297638	1.514842	1.823034
O	-1.544960	2.727527	2.462308
H	1.871264	-2.363515	-2.670433
H	5.037987	-3.172434	0.127478
H	4.215419	-1.349831	1.562323
C	3.513089	-2.896546	-1.368234
C	3.702691	-1.559567	0.633322
N	2.325635	2.552592	-1.998783
H	1.885552	1.636464	-1.995822
C	3.579852	2.480843	-1.264456
H	4.189717	1.659547	-1.654324
H	4.142283	3.414622	-1.398508
H	3.445455	2.322464	-0.179293
C	1.398253	3.522592	-1.427173
H	1.785051	4.538757	-1.581999
H	0.431399	3.445820	-1.932427
H	1.229021	3.384783	-0.345492
